# Transitions in oral and gut microbiome of HPV+ oropharyngeal squamous cell carcinoma following definitive chemoradiotherapy (ROMA LA-OPSCC study)

**DOI:** 10.1038/s41416-020-01253-1

**Published:** 2021-03-10

**Authors:** Marc Oliva, Pierre H. H. Schneeberger, Victor Rey, Matthew Cho, Rachel Taylor, Aaron R. Hansen, Kirsty Taylor, Ali Hosni, Andrew Bayley, Andrew J. Hope, Scott V. Bratman, Jolie Ringash, Simron Singh, Ilan Weinreb, Bayardo Perez-Ordoñez, Douglas Chepeha, John Waldron, Wei Xu, David Guttman, Lillian L. Siu, Bryan Coburn, Anna Spreafico

**Affiliations:** 1 Division of Medical Oncology and Hematology, Princess Margaret Cancer Centre, University of Toronto, Toronto, ON Canada; 2 Department of Medical Oncology, Catalan Institute of Oncology (ICO), Barcelona, Spain; 3 Division of Infectious Diseases and Toronto General Hospital Research Institute, University Health Network, Departments of Medicine, Immunology and Laboratory of Medicine and Pathobiology, University of Toronto, Toronto, ON Canada; 4 Department of Radiation Oncology, Princess Margaret Cancer Centre, University of Toronto, Toronto, ON Canada; 5 Department of Pathology, Princess Margaret Cancer Centre, University of Toronto, Toronto, ON Canada; 6 Department of Medical Oncology, Sunnybrook Health Sciences Centre, Toronto, ON Canada; 7 Department of Surgical Oncology, Princess Margaret Cancer Centre, University of Toronto, Toronto, ON Canada; 8 Department of Biostatistics, Princess Margaret Cancer Centre, University of Toronto, Toronto, ON Canada; 9 Department of Cell and Systems Biology, University of Toronto, Toronto, ON Canada

**Keywords:** Cancer microenvironment, Oral cancer

## Abstract

**Background:**

Oral and gut microbiomes have emerged as potential biomarkers in cancer. We characterised the oral and gut microbiomes in a prospective observational cohort of HPV+ oropharyngeal squamous cell carcinoma (OPSCC) patients and evaluated the impact of chemoradiotherapy (CRT).

**Methods:**

Saliva, oropharyngeal swabs over the tumour site and stool were collected at baseline and post-CRT. 16S RNA and shotgun metagenomic sequencing were used to generate taxonomic profiles, including relative abundance (RA), bacterial density, α-diversity and β-diversity.

**Results:**

A total of 132 samples from 22 patients were analysed. Baseline saliva and swabs had similar taxonomic composition (*R*^2^ = 0.006; *p* = 0.827). Oropharyngeal swabs and stool taxonomic composition varied significantly by stage, with increased oral RA of *Fusobacterium nucleatum* observed in stage III disease (*p* < 0.05). CRT significantly reduced the species richness and increased the RA of gut-associated taxa in oropharyngeal swabs (*p* < 0.05), while it had no effect in stool samples. These findings remained significant when adjusted by stage, smoking status and antibiotic use.

**Conclusions:**

Baseline oral and gut microbiomes differ by stage in this HPV+ cohort. CRT caused a shift towards a gut-like microbiome composition in oropharyngeal swabs. Stage-specific features and the transitions in oral microbiome might have prognostic and therapeutic implications.

## Background

The human microbiome has recently emerged as a promising biomarker in cancer.^1^ The microbiome inhabiting the oro-gastrointestinal tract has been implicated in the carcinogenesis of many tumour types and in modulating responses to anti-cancer therapies, including immunotherapy, although the mechanisms are not yet well understood.^2–4^ A few studies have shown differential oral microbial composition in the saliva of patients with oral cavity and oropharyngeal tumours when compared to healthy individuals, while specific commensals have been associated with lower risk of developing head and neck squamous cell carcinomas (HNSCC).^5–7^ Oral microbiome composition seems to vary across different primary sites (e.g. oral cavity vs oropharynx) or according to stage, human papillomavirus (HPV) status and treatment received (e.g. surgery vs chemoradiotherapy (CRT)), suggesting a role as a tumour-specific biomarker in this disease, with potential impact on treatment efficacy and toxicity.^8–10^ However, the evaluation of the oral microbiome in HNSCC has thus far been limited to retrospective and heterogeneous cohorts of patients, while the gut microbiome is yet to be investigated.

Among HNSCC, the incidence of oropharyngeal squamous cell carcinoma (OPSCC) has dramatically increased over the past decade, with HPV-related disease being most prevalent.^11,12^ HPV-positive (HPV+) OPSCC are a biologically distinct disease with increased treatment responsiveness and survival when compared to HPV-negative tumours.^13^ As such, multiple studies are evaluating de-escalation strategies in the locoregionally advanced (LA) setting to reduce treatment toxicity without compromising survival.^14^ However, HPV+ tumours are heterogeneous and not all have a favourable prognosis.^15^ Beyond clinical and pathological factors such as smoking history, tumour, node, metastasis (TNM) staging and HPV status, there remains an unmet need for new biomarkers that provide accurate risk stratification of this patient population. In this regard, HPV+ LA-OPSCC represents a unique setting to evaluate and compare both tumour-associated and gut microbiomes and their potential effect on treatment.

ROMA LA-OPSCC is the first study to prospectively characterise both oral and gut microbiomes and to evaluate the impact of definitive CRT on their composition in a homogeneous cohort of newly diagnosed HPV+ LA-OPSCC.

## Methods

### Patient population and study design

ROMA LA-OPSCC (NCT03759730) is a single-centre, non-interventional, investigator-initiated feasibility study designed to evaluate the oral and intestinal microbiome in a prospective cohort of patients with HPV+ LA-OPSCC treated with definitive CRT. Patients with previously untreated histologically proven OPSCC (tonsil, base of tongue, soft palate) candidates for definitive concurrent CRT with single-agent cisplatin (CDDP) as per standard of care were eligible. HPV status was determined by p16 immunohistochemical staining and classified as positive if nuclear and cytoplasmic staining in ≥70% tumour cells. In situ hybridisation to confirm the presence of high-risk HPV DNA was performed in equivocal cases. All patients were staged and treated according to eighth edition TNM staging criteria. Treatment and follow-up assessments were conducted according to institutional protocol (Supplement). Saliva, oropharyngeal swabs over the tumour site and stools samples were collected before treatment (up to 3 weeks prior to the start of radiotherapy) and at completion of CRT (up to 3 weeks following last day of radiotherapy; Supplementary Fig. 1). Patients were evaluable for analysis if samples were provided at least at one time point. The study was approved by the institutional research ethics board. All patients provided written, signed, informed consent to participate.

### Treatment and follow-up

All patients received intensity-modulated radiotherapy to a gross tumour dose of 70 Gy in 35 fractions over 7 weeks (2 Gy/fraction). Concurrent CDDP (three-weekly at 100 mg/m^2^ on RT days 1, 22 and 43 or weekly at 40 mg/m^2^ for 7 weeks) was delivered according to institutional protocol. The choice of a three-weekly versus weekly schedule was based on patient’s Eastern Cooperative Oncology Group Performance scale and comorbidities as assessed by medical oncologist. All patients had a prophylactic gastrostomy tube placed within 3–4 weeks from the start of radiation as per institutional standard practice. Follow-up after treatment completion was conducted according to institutional protocol. Local and regional recurrences were confirmed histologically, while distant metastases were diagnosed by unequivocal clinical/radiologic evidence +/− histologic confirmation. Clinical data were abstracted prospectively (M.O.) for all patients enrolled in the study.

### Sample collection and microbiome analysis

Saliva, oropharyngeal swab over the tumour site and stool samples were collected using the ZymoBIOMICS DNA/RNA Mini Prep^TM^ kits (Zymo Research, Irvine, CA). Sampling and storage protocol are available as Supplementary Data (Laboratory Manual). Processing and analysis of the samples was conducted at the Centre for Genome Evolution and Function (CAGEF) of the University of Toronto. DNA was extracted using the ZymoBIOMICS DNA Micro Kit^TM^. 16S rRNA gene sequencing (Supplement) was performed on saliva (*n* = 46), oropharyngeal swabs (*n* = 46) and stool samples (*n* = 46). Briefly, the V4 hypervariable region of the 16S rRNA gene is amplified using an universal forward sequencing primer and a uniquely barcoded reverse sequencing primer to allow for multiplexing.^16^ Amplicon sequencing was performed on an Illumina MiSeq platform (Illumina, CA, USA) with V2 chemistry as described in Schneeberger et al.^17^. Taxonomic profiling of 16S data sets was performed using the UNOISE pipeline.^18^ Shotgun metagenomics sequencing was only performed on oropharyngeal swabs (*n* = 46) and stool samples (*n* = 46). Libraries were constructed using the Illumina Nextera Flex kits (Illumina, USA) using 150 ng DNA as input. A total of 1.94 Billion reads were generated on an Illumina NovaSeq 6000 platform (Illumina, USA) using a SP flow cell and reagents according to the manufacturer’s protocol at the Princess Margaret Genomics Centre. A median of 2.3E + 07 [1.35E + 07–4.01E + 07] reads for stool samples and of 9.16E + 05 [2.17E + 05–3.09E + 07] reads for oropharyngeal samples were remaining after host read removal with Kneaddata v. 0.7.2 (https://bitbucket.org/biobakery/biobakery/wiki/kneaddata). Taxonomic profiles resulting from shotgun datasets were generated using Metaphlan2 with the Chocophlan database v. 293.^19,20^ Alpha diversity and beta diversity were measured using the Phyloseq package (ref. ^20^; v. 3.9) and VEGAN v. 2.5.5^21^ in R v. 3.5.3.^22^

### Statistical analysis

ROMA LA-OPSCC is a signal-finding study. Descriptive statistics were used to summarise clinical and microbiome characteristics. Mixed model regression was conducted to explore the potential demographic and clinical factors that are related to the microbial change during CRT. For microbiome analyses, summary statistics were described including within-patient community composition (taxonomic relative abundance), alpha diversity (compositional diversity within-sample) using Shannon index (SDI; a composite metric of both richness and evenness) and Berger–Parker index (BP; an indicator of dominance in the community), as well as beta-diversity (inter-sample similarity) of baseline and end of treatment samples. Alpha diversity measures were compared between groups using Mann–Whitney (MW) tests. LEFSE was used to measure the differences in relative abundances (non-parametric Kruskal–Wallis tests) and the effect size (linear discriminant analysis) between groups. Beta diversity was measured using the Bray–Curtis dissimilarity index and group comparisons were conducted using permutational multivariate analysis of variance (PERMANOVA). Interaction between treatment effect (changes in composition pre- and post-CRT) and other variables including use of antibiotics, G-tube dependency, grade of mucositis, smoking status, tumour location, stage and T staging were measured using PERMANOVA. Assuming a significance level for alpha of 0.01 to adjust for multiple comparisons of key taxa, alpha diversity, and beta diversity, our study with 22 patients’ microbiome samples had at least 85% power to identify significant differences between pre- and post-CRT, given an effect size of 0.7 standard deviation (SD) of the paired mean difference. The power analysis is based on two-sided paired *t* tests.

## Results

### Clinical characteristics and outcome

From January 2018 to November 2018, 26 patients with newly diagnosed LA-OPSCC candidates for CRT were enrolled in the study, of which 22 were included in this analysis. Four were excluded for reasons outlined in the Consort diagram (Supplementary Fig. 2). Patient characteristics are summarised in Supplementary Table 1. Most patients were male and smokers (current or former) with ≥10 pack-year smoking history. Thirty-six percent of patients had stage III disease at presentation with tonsil being the most common primary site. Eleven patients received antibiotics up to 1 month prior to and/or during CRT. At the time of data cut-off, with a median follow-up of 90 weeks (20–115), all patients were alive and 21/22 were disease free. One patient (R05) developed locoregional and distant recurrence.

### Description of baseline oral and stool microbiome in HPV+ LA-OPSCC

**Fig. 1:**
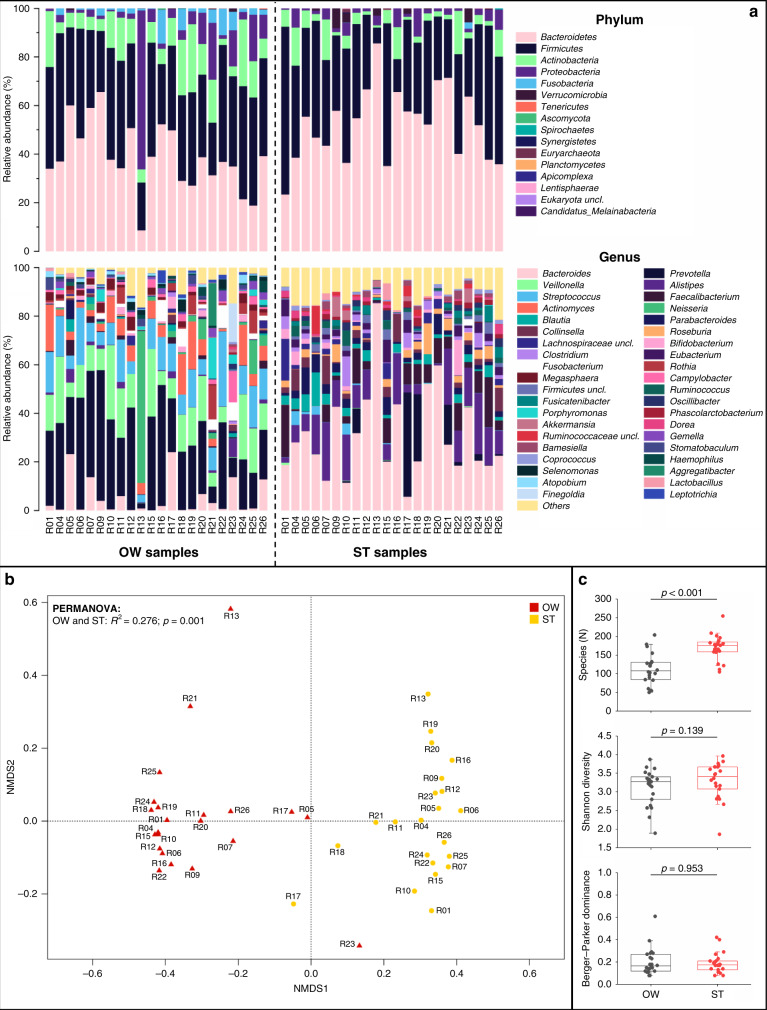
Taxonomic composition differed by sampling site (oropharyngeal swabs vs stool samples). **a** Community composition of oral microbiome at the phylum and genus levels. Species-level composition is summarised in Supplementary Table 4. **b** Non-metric multidimensional scaling plot comparing taxonomic composition of sample types at baseline. **c** Alpha diversity indices in stool and oral communities, including identified species (upper panel), Shannon diversity (central panel), and Berger–Parker dominance (lower panel). OW oropharyngeal swabs over the tumour site, ST stools.

A total of 132 samples collected from the 22 evaluable patients (100% compliance in sample acquisition) were analysed. Taxonomic composition of oropharyngeal swabs and saliva samples by 16S rRNA gene sequencing were similar (*R*^2^ = 0.06; *p* = 0.827; Supplementary Fig. 3), thus shotgun metagenomic sequencing was only conducted in oropharyngeal swabs and stool. All subsequent results are based on shotgun metagenomic sequencing analyses. Taxonomic composition differed by sampling site (oropharyngeal swabs vs stool samples: *R*^2^ = 0.276; *p* = 0.001; Fig. 1a, b). Oral communities comprised mostly oropharyngeal anaerobes and facultative anaerobes, including *Prevotella*, *Veillonella*, *Streptococcus* and *Actinomyces* species while stool communities were composed mainly of obligate anaerobic *Bacteroides* species. The number of species was higher in the stool vs oral communities (*p* < 0.0001) but they had overall similar diversity (SDI_mean_ = 3.3 for stool and 3.12 for oropharyngeal samples; BP_mean_ = 0.19 for stool and 0.2 for oropharyngeal samples; Fig. 1c). Four patients (R05, R17, R23 and R26) had a high proportion (>10% of the community) of *Bacteroides* species in their oropharyngeal swabs more typical of the lower intestinal tract.

### Differential baseline oral and stool microbiome composition by stage

**Fig. 2:**
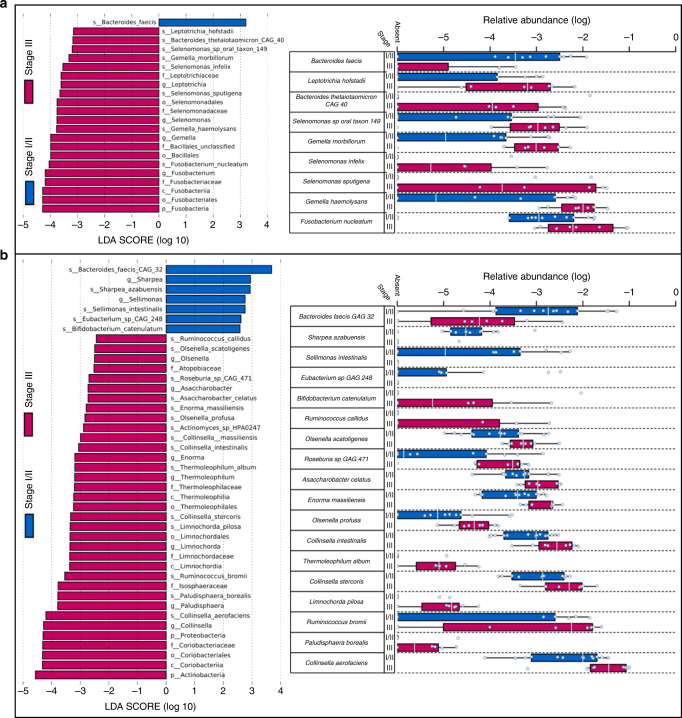
Compositional differences in the oral and intestinal communities differed by disease stage. **a** Compositional differences in the oral communities at baseline. The left panel indicates taxonomic features (at all taxonomic levels) different in abundance at early (stage I/II) and advanced (stage III) disease stage (LDA > 2.0; *p* < 0.05). The right panel shows the difference in normalised relative abundance of enriched/depleted species. **b** Compositional differences in the gut communities. The left panel highlights all differentially abundant taxonomic features between early and advanced disease stage (LDA > 2.0; *p* < 0.05). The right panel shows the difference in normalised relative abundance of identified species. The LDA score indicates the effect size of the differences observed between groups.

Taxonomic composition of oropharyngeal swabs significantly differed across stage III vs stage I–II patients (*p* < 0.05): four genera were enriched in patients with stage III, including *Fusobacterium* (*Fusobacterium nucleatum*), *Gemella* (*Gemella morbillorum* and *Gemella haemolysans*), *Leptotrichia* (*Leptotrichia hofstadii*) and *Selenomonas* (*Selenomonas sputigena* and *Selenomonas infelix*) (Fig. 2a). Taxonomic composition of stool samples also differed in stage III vs stage I–II disease, with significant enrichment of two phyla, *Actinobacteria* and *Proteobacteria*, and 18 species (*p* < 0.05; Fig. 2b).

In the univariate analysis, no effect on baseline oropharyngeal swab microbiome composition was seen by smoking or primary tumour location, although a trend was observed by T staging (*p* = 0.06; Supplementary Table 2).

### Impact of CRT on the oral and stool microbiome

#### Oral microbiome

**Fig. 3:**
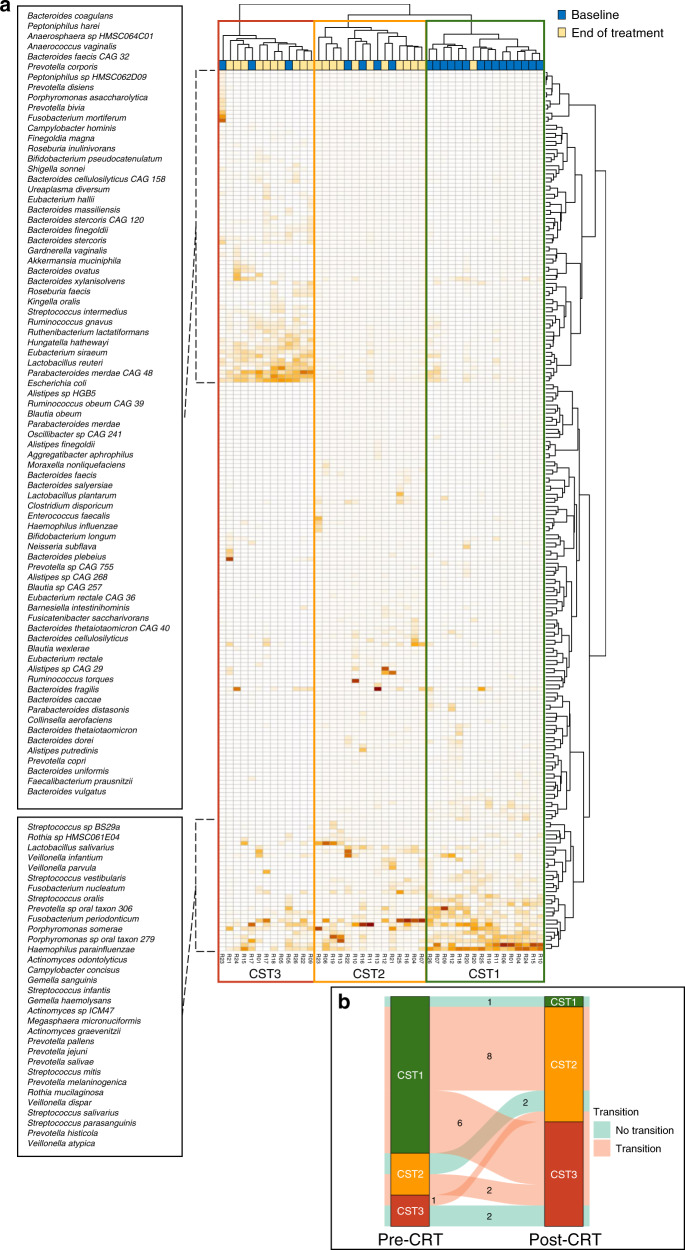
Compositional differences in oral communities based on collection time point (baseline vs post-CRT). **a** Taxonomic composition of pre-/post-treatment oral microbiome clustered using Bray–Curtis dissimilarity (columns) and Ward’s clustering method (rows). **b** Community transitions observed in oropharyngeal swabs after CRT (paired samples). The number of patients which transition from one community to another after CRT is indicated with each arrow. CRT chemoradiotherapy.

We compared the composition of oropharyngeal swabs pre- and post-CRT (Fig. 3). We observed the formation of three distinct clusters based on collection time point baseline vs post-CRT (Fig. 3a): cluster 1 (15 baseline vs 1 post-CRT samples) was characterised by high relative abundance of species from the *Veillonella*, *Prevotella* and *Streptococcus* genera; cluster 2 (4 baseline vs 11 post-CRT samples) was characterised by high abundances of *Streptococcus* species, *Prevotella melaninogenica*, *Neisseria flavescens* and *Rothia mucilaginosa*, among others; and cluster 3 (3 baseline vs 10 post-CRT samples) was characterised by high abundances of species from the *Bacteroides*, *Faecalibacterium*, *Prevotella* (*Prevotella copri*), *Collinsella*, *Alistipes* and *Parabacteroides* genera. Overall, the number of species was significantly reduced in post-CRT oropharyngeal swabs (MW; *p* = 0.006). Alpha diversity did not change post-CRT (SDI_mean_ = 3.12 at baseline and 3.09 at the end of treatment; BP_mean_ = 0.2 at baseline and 0.2 at the end of treatment; MW; *p*_SDI_ = 0.716; *p*_BP_ = 0.944) nor did bacterial density (8.8E + 09 16S copies/ml at baseline and 2.6E + 09 16S copies/ml at the end of treatment; *p* = 0.15) (Supplementary Fig. 4). Intra-patient changes in community composition post-CRT are summarised in Fig. 3b. Most patients (65%) were classified in cluster 1 at baseline while only 1 patient (4.5%) was classified in cluster 1 post-CRT. Out of the 15 patients in cluster 1 at baseline, 8 transitioned to cluster 2 and 6 to cluster 3 post-CRT. None of the patients who grouped in cluster 2 and 3 at baseline shifted to cluster 1 after CRT and remained within the cluster 2 or 3.

Clinical characteristics associated with cluster subgroups are summarised in Table 1. Out of the 15 patients grouping in cluster 1 at baseline, the majority were former/non-smokers (93%), with tonsillar primary (67%) and stage I–II disease (67%). Baseline oral composition from 3 out of the 4 current smokers of the cohort belonged to cluster 2 or 3. No clear pattern was seen between cluster transitions post-CRT by TNM, stage, smoking status, use of antibiotics, grade of mucositis or gastrostomy tube dependency post-CRT. One of the two patients (patient R05) with cluster 3-type oral microbial composition at baseline and post-CRT experienced biopsy-proven locoregionally and distant recurrence.

**Table 1:** Patient baseline and post-CRT community state types matched with clinical characteristics.

Patient ID	BSL cluster	EOT cluster	Tumour location	TNM	Stage	Smoking status	Antibiotic use	Grade of mucositis	Gastrostomy dependancy^a^
R01	1	3	Tonsil	T3N2b	II	Current	No	3	Yes
R04	1	2	Base of tongue	T1N3	III	Never	Yes	3	UK
R06	1	2	Tonsil	T3N2	II	Never	No	2	Yes
R07	1	2	Tonsil	T2N3	III	Former	Yes	2	Yes
R09	1	3	Tonsil	T2N1	I	Never	Yes	2	Yes
R10	1	2	Tonsil	T4N1	III	Never	No	2	Yes
R11	1	2	Tonsil	T3N1	II	Former	Yes	3	Yes
R12	1	2	Base of tongue	T2N1	I	Former	No	2	Yes
R15	1	3	Base of tongue	T2N1	I	Former	Yes	3	No
R18	1	3	Base of tongue	T4N1	III	Former	Yes	2	Yes
R19	1	2	Tonsil	T2N1	I	Former	Yes	1	No
R20	1	1	Soft palate	T1N1	I	Never	Yes	1	No
R24	1	3	Tonsil	T3N1	II	Never	No	3	Yes
R25	1	2	Base of tongue	T1N1	I	Never	No	1	No
R13	2	2	Base of tongue	T3N1	II	Never	Yes	2	No
R16	2	2	Tonsil	T4N0	III	Current	No	2	Yes
R21	2	3	Tonsil	T4N0	III	Current	No	2	Yes
R22	2	3	Soft palate	T4N1	III	Former	No	3	Yes
**R05**	3	3	Tonsil	T1N2c	II	Current	Yes	2	Yes
R17	3	3	Tonsil	T3N0	II	Former	Yes	2	Yes
R23	3	2	Base of tongue	T1N2	II	Former	No	2	No

Patient R05 (in bold) is the only patient in the cohort who experience disease recurrence.

*BSL* baseline, *EOT* end of treatment.

^a^G-tube dependency at the time of collection of EOT samples, up to 3 weeks from the last day of RT.

**Fig. 4:**
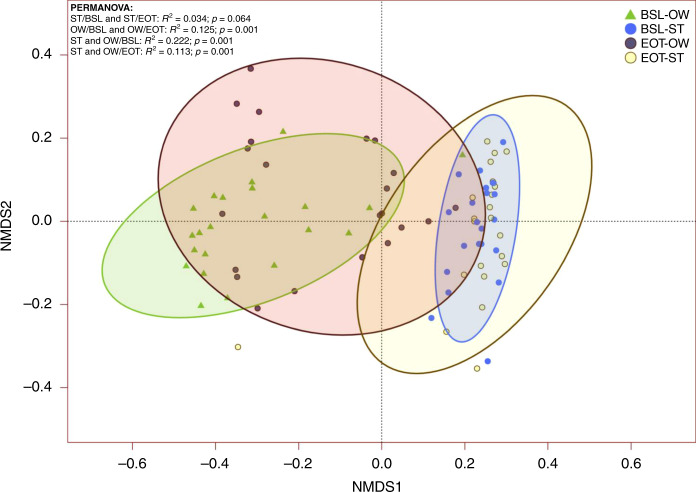
Impact of chemoradiation therapy on oral and intestinal microbial communities. Non-metric multidimensional scaling ordination plot based on Bray–Curtis dissimilarity with group-specific standard deviational ellipse (90%). CRT chemoradiation, BSL baseline, EOT end of treatment, OW oropharyngeal swabs over the tumour site.

Overall, the compositional changes between baseline and post-CRT consisted of a shift towards gastrointestinal tract-like communities (Fig. 4). Oropharyngeal swabs post-CRT clustered closer to stool samples, with significant changes in taxa composition when compared to baseline (*R*^2^ = 0.1; *p* = 0.001). The dissimilarity observed between oropharyngeal swabs and stool samples was reduced in post-CRT samples (PERMANOVA; *R*^2^ = 0.115; *p* = 0.001) compared to baseline samples (PERMANOVA; *R*^2^ = 0.203; *p* = 0.001). Functional analyses associated with these taxonomic findings were attempted but could not be performed due to insufficient sequencing depth (Supplementary Fig. 5).

#### Stool microbiome

No clustering was observed in stool samples by collection time point (baseline vs post-CRT); similar taxa composition and alpha diversity was observed post-CRT (Supplementary Fig. 6). No differences in taxonomic composition were observed in post-treatment samples based on the use of antibiotics (Supplementary Table 3 and Supplementary Fig. 7).

The impact of CRT (defined as collection time point: baseline and post-CRT) on the oral microbiome remained significant when adjusting by potential confounding factors, including smoking status, TNM, stage subgroups, maximum grade of mucositis, gastrostomy tube dependency 3–4 weeks post-CRT and use of antibiotics (Supplementary Table 4).

## Discussion

ROMA LA-OPSCC is the first study to prospectively characterise both oral and gut microbiomes in HPV+ OPSCC patients treated with definitive CRT. We found that both oral and stool community composition differed by disease stage at baseline and that the oral but not stool microbiome composition changed after CRT. The shift in oropharyngeal taxonomic composition after treatment was largely driven by an increase in the relative abundance of gut-associated obligate anaerobes. The results of this study provide a step forward in the understanding of both microbiomes in this disease and may be used as a benchmark as new treatments are being investigated in this patient population.

The composition of the oral microbiome in our cohort was comparable to that of other retrospective cohorts involving patients with oral cavity and oropharyngeal tumours.^8,23^ Guerrero-Preston et al. reported differential taxonomic composition in HPV+ OPSCC when compared to HPV-negative OPSCC and oral cavity cancer, with higher prevalence of *Veillonella*, *Prevotella*, *Streptococcus* and *Gemella* genera.^8,9^ In our cohort involving HPV+ disease exclusively, we did observe a similar taxonomic composition at the genus level. Our shotgun metagenomics analysis revealed differential oral microbial composition across stages, and patients with stage III had significantly higher relative abundance of *F. nucleatum* species. *F. nucleatum* had been previously described in heterogeneous cohorts involving HNSCC patients treated with surgery and/or radiation, but it has also been recently associated with advanced disease, chemotherapy resistance and adverse prognosis in other tumour types, such as oesophageal carcinoma.^9,24–26^ In our cohort, the stage differences in oral composition seemed to be associated with larger primary tumours. Patients with stage III HPV+ OPSCC are known to be at higher risk of recurrence despite definitive concurrent chemoradiation and new treatment intensification approaches including immunotherapy that are being explored in this setting (e.g. NCT03040999).^27^ It remains to be tested whether these findings have prognostic implications and therefore could be used for risk stratification in this patient population. Interestingly, while smoking history seems to have a role as prognostic biomarker for HPV+ disease and has also been highlighted to correlate with oral dysbiosis, we did not observe differences in oral microbiome composition according to smoking status in our overall cohort or by stage.^28–30^

We evaluated the changes post-CRT on both oral and stool microbiomes. Two studies involving patients with HNSCC and nasopharyngeal carcinoma have described changes in the oral microbiota following radiation and an increase in opportunistic pathogens.^31,32^ Our analysis revealed a significant and consistent impact of CRT in the overall oral communities among the cohort, with increases in the prevalence and relative abundance of obligate anaerobes (e.g. *Bacteroides* species). The cause of these shifts is unclear but may be due to treatment-induced tissue necrosis or other changes in the tumour-adjacent mucosa, direct effects of CRT on the microbes themselves or treatment-associated immune or metabolic changes in the local tissues affecting microbial ecology. The potential biological and/or clinical impact of baseline and post-treatment composition or shifts after CRT remains unknown and long-term follow-up is required. Of note, one of the two patients harbouring a “gut-like” oropharyngeal taxa both at baseline and post-CRT experienced disease recurrence about a year after treatment completion.

We did not observe any significant shift in the gut microbiome composition after CRT in our cohort. While radiation is a local therapy and thus it is not expected to specifically alter the gut microbiome, cytotoxic chemotherapeutic agents including cisplatin are known to induce damage of the intestinal mucosa and disrupt the microbiome, leading to increased risk of infections.^33^ The heterogeneity of gut microbiome composition at baseline and the limited number of patients might have limited the detection of differences due to CRT or antibiotic use. Although there were intra-patient changes in gut microbiome composition in our study, these changes were patient specific and no common pattern was observed in the overall cohort.

There were no differences in the overall taxa composition between saliva and oropharyngeal swabs taken from the tumour site. This is particularly relevant as this patient population is characterised by radiation-induced xerostomia,^34^ and thus the swab could substitute the collection of saliva, the collection of which can be a challenge following completion of CRT in this patient population. Zhang et al. reported differential taxa composition between saliva and tumour tissue from patients with oral cavity tumours, with significantly higher levels of *F. nucleatum* and *Acinetobacter* found in the tumour.^25^ Whether microbiome data that are obtained from oropharyngeal swabs differ from those from tumour tissue was not assessable in our study.

The limitations of our study include: inability to account for all patient factors that may influence oral and stool microbial community composition, such as dietary habits and dental hygiene; short median follow-up for HPV+ OPSCC disease limiting the evaluation of the prognostic impact of microbiome signatures; lack of further sampling beyond 4 weeks from CRT limiting the evaluation of long-term oral and stool microbiome alterations post-CRT^31,35^; small number of patients involved, which prevents statistical power for specific subgroup analysis. We used both 16S rRNA and shotgun sequencing techniques for two reasons. We first wanted to assess the level of agreement between different samples types retrieved in the same body compartment (saliva vs oropharyngeal swabs). For this exploratory analysis, 16S sequencing is sufficiently sensitive to compare the overall composition between sample types with a relatively low cost. Based on the high agreement between both sample types, we then selected oropharyngeal swabs for shotgun sequencing, as it has higher taxonomic resolution to observe CRT-mediated changes at the species level. This combination of approaches allowed us to gain the greatest amount of high-resolution microbiome compositional data at the lowest cost. A ‘shallow’ shotgun approach was used to characterise the taxonomic composition in the different sample types, which was sufficient for the detection of species above relative abundance of 0.05% but it did not allow us to conduct functional analyses.^36^

This pilot study shows that prospective characterisation of both oral and stool microbiome is feasible in this patient population, with 100% compliance in sample acquisition and analysis. The stage-specific microbial features in the oral and gut communities from this cohort are hypothesis-generating and should be further investigated to evaluate their use as a biomarker for risk stratification in patients with HPV+ OPSCC. Additional correlation with HPV-related factors such as serotype or viral load in saliva and comparison with a matched-HPV-negative cohort are to be explored. These findings might serve as a ‘control’ for the microbiome landscape as therapeutic interventions such as immunotherapy are being incorporated into the treatment of these patient populations. Indeed, prospective evaluation of oral and intestinal microbiome is currently ongoing in the setting of an international prospective chemo-sparing approach evaluating definitive chemoradiation vs immunoradiotherapy in HPV+ intermediate-risk OPSCC (NCT03410615). The transitions observed in the composition of the oral but not gut microbiome following treatment might not only have prognostic value but also therapeutic implications to explore gut microbiome modulation strategies in this setting. In this regard, we are currently evaluating the feasibility of gut microbiome intervention in the context of CRT in patients with LA-OPSCC using an oral consortium of taxa associated with immune checkpoint inhibitor-responsiveness (NCT03838601).

## Supplementary information

Supplementary Material

Taxonomic profiles

## Data Availability

The data sets generated and/or analysed during the current study are not yet publicly available but are available from the corresponding author on reasonable request.
