# Supplementary material for: Transitions in oral and gut microbiome of HPV+ oropharyngeal squamous cell carcinoma following definitive chemoradiotherapy (ROMA LA-OPSCC study)
Source: Br J Cancer. 2021 Mar 10;124(9):1543–51. doi: 10.1038/s41416-020-01253-1 (PMC8076306; doi:10.1038/s41416-020-01253-1)
Supplement: Supplementary file 1 — Supplementary Material [file 41416_2020_1253_MOESM1_ESM.pdf]

Supplemental information belonging to:

Transitions in oral and gut microbiome of HPV+ oropharyngeal squamous cell carcinoma following definitive chemoradiotherapy (ROMA LA-OPSCC study)

Article type: Original Article/Research.

Authors: Marc Oliva[1*] and Pierre H.H. Schneeberger[2*], Victor Rey[2], Rachel Taylor[1], Aaron R. Hansen[1], Kirsty Taylor[1], Andrew Bayley[3], Andrew J Hope[3], Ali Hosni Abdalaty[3], Scott V. Bratman[3], Jolie Ringash[3], Ilan Weinreb[4], Bayardo Perez-Ordonez[4], Ralph Gilbert[5], John Waldron[3], Wei Xu[6], David Guttman[7], Lillian L. Siu[1], Bryan Coburn[2**] and Anna Spreafico[1**].

* These two authors contributed equally.

** Both

Affiliations:

[1]*Division of Medical Oncology and Hematology, Princess Margaret Cancer Centre, University of Toronto, Canada.*

[2]*Division of Infectious Diseases, University Health Network, Departments of Medicine and Laboratory of Medicine and Pathobiology, University of Toronto, Toronto, Canada.*

[3]*Department of Radiation Oncology, Princess Margaret Cancer Centre, University of Toronto, Canada.*

[4]*Department of Pathology, Princess Margaret Cancer Centre, University of Toronto, Canada.*

[5]*Department of Surgical Oncology, Princess Margaret Cancer Centre, University of Toronto, Canada.*

[6]*Department of Biostatistics, Princess Margaret Cancer Centre, University of Toronto, Canada.*

[7]*Department of Cell and Systems Biology, University of Toronto, Canada.*

Supplementary Figure Captions

Supplementary Figure 1. ROMA LA-OPSCC study design.

Supplementary Figure 2. Flow diagram of patients enrolled and included in the analysis.

Supplementary Figure 3. **Oral microbiome composition using saliva vs Oropharyngeal swabs. A**. Compositional dissimilarity between saliva and oropharyngeal samples based on 16S rRNA gene sequencing. **B**. Intra-individual diversity compared to inter-patient diversity using Bray-Curtis dissimilarity as a distance measure.

Supplementary Figure 4. Impact of chemoradiation therapy on oral and intestinal microbial diversity. Alpha diversity indices (upper left, lower left and right panels) and bacterial density (upper right panel) comparison in oral communities at baseline and post-CRT.

Supplementary Figure 5. Effect of sequencing depth on various indices used for taxonomic as well as functional profiling of microbial communities. A-E. Scatter plot showing the associations between several metrics used for taxonomic and functional analyses and sequencing depth. **F.** Spearman correlation coefficient and statistical significance between sequencing depth and taxonomic and functional metrics. A lack of correlation indicates that there is no bias introduced by sequencing depth whereas a correlation indicates that results are confounded by sequencing depth.

Supplementary Figure 6. Effect of chemoradiation therapy (CRT) on the intestinal microbiome. A. Heatmap showing sample taxonomic composition and clustering based on Bray-Curtis dissimilarity. **B.** Alpha diversity indices stratified by collection time, including species number (left panel), Shannon diversity (middle panel), and Berger-Parker dominance (right panel). The *P*-value is calculated using a non-parametric Wilcoxon test.

Supplementary Figure 7. Gut communities in post-treatment stool samples based on the use of antibiotics and the route of administration.

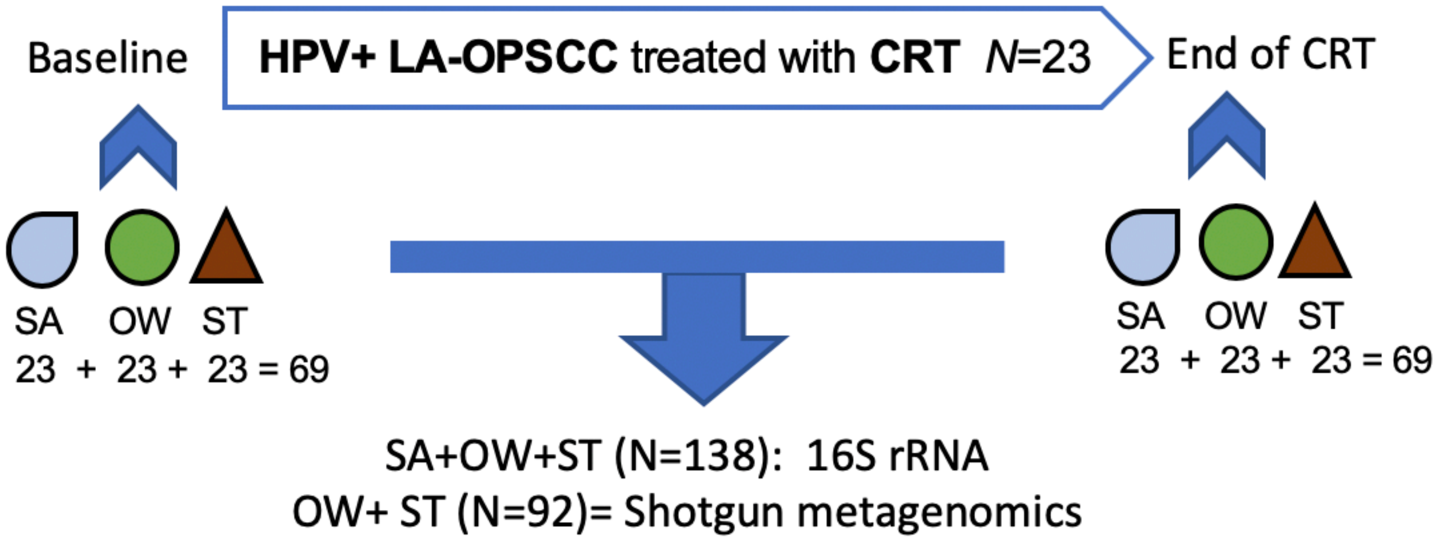
Baseline
HPV+ LA-OPSCC treated with CRT N=23
End of CRT
SA OW ST
23 + 23 + 23 = 69
SA OW ST
23 + 23 + 23 = 69
SA+OW+ST (N=138): 16S rRNA
OW+ ST (N=92)= Shotgun metagenomics

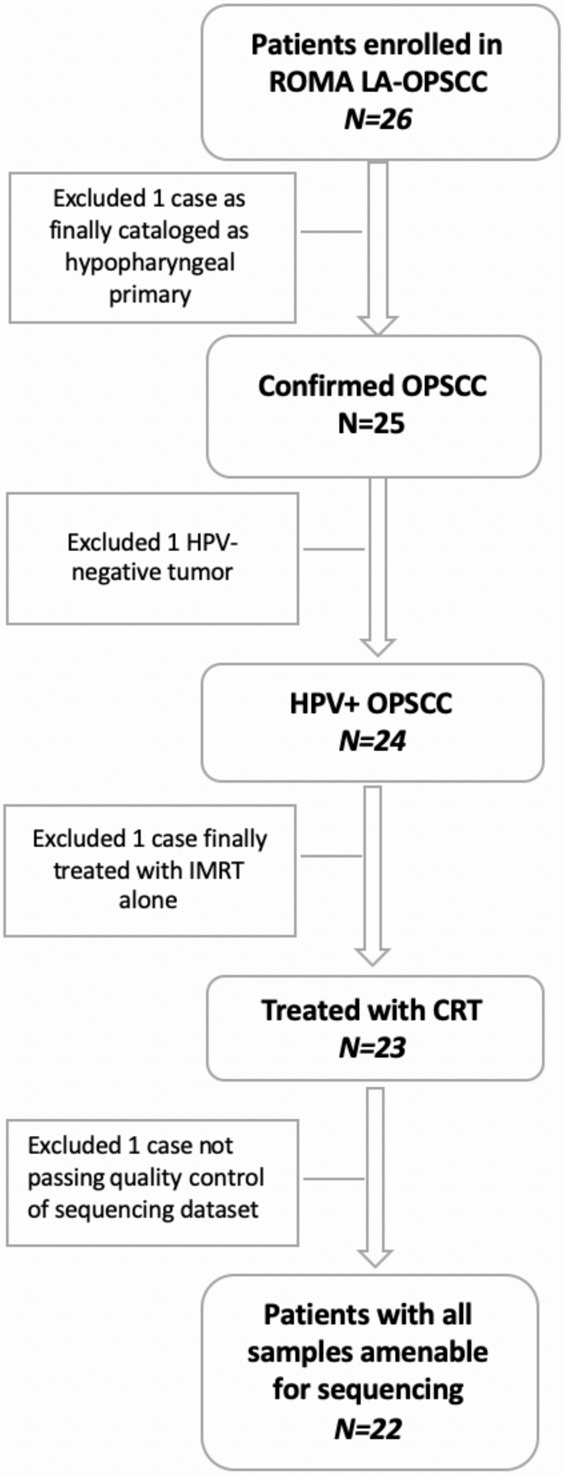
Patients enrolled in ROMA LA-OPSCC
N=26
Excluded 1 case as finally cataloged as hypopharyngeal primary
Confirmed OPSCC
N=25
Excluded 1 HPV-negative tumor
HPV+ OPSCC
N=24
Excluded 1 case finally treated with IMRT alone
Treated with CRT
N=23
Excluded 1 case not passing quality control of sequencing dataset
Patients with all samples amenable for sequencing
N=22

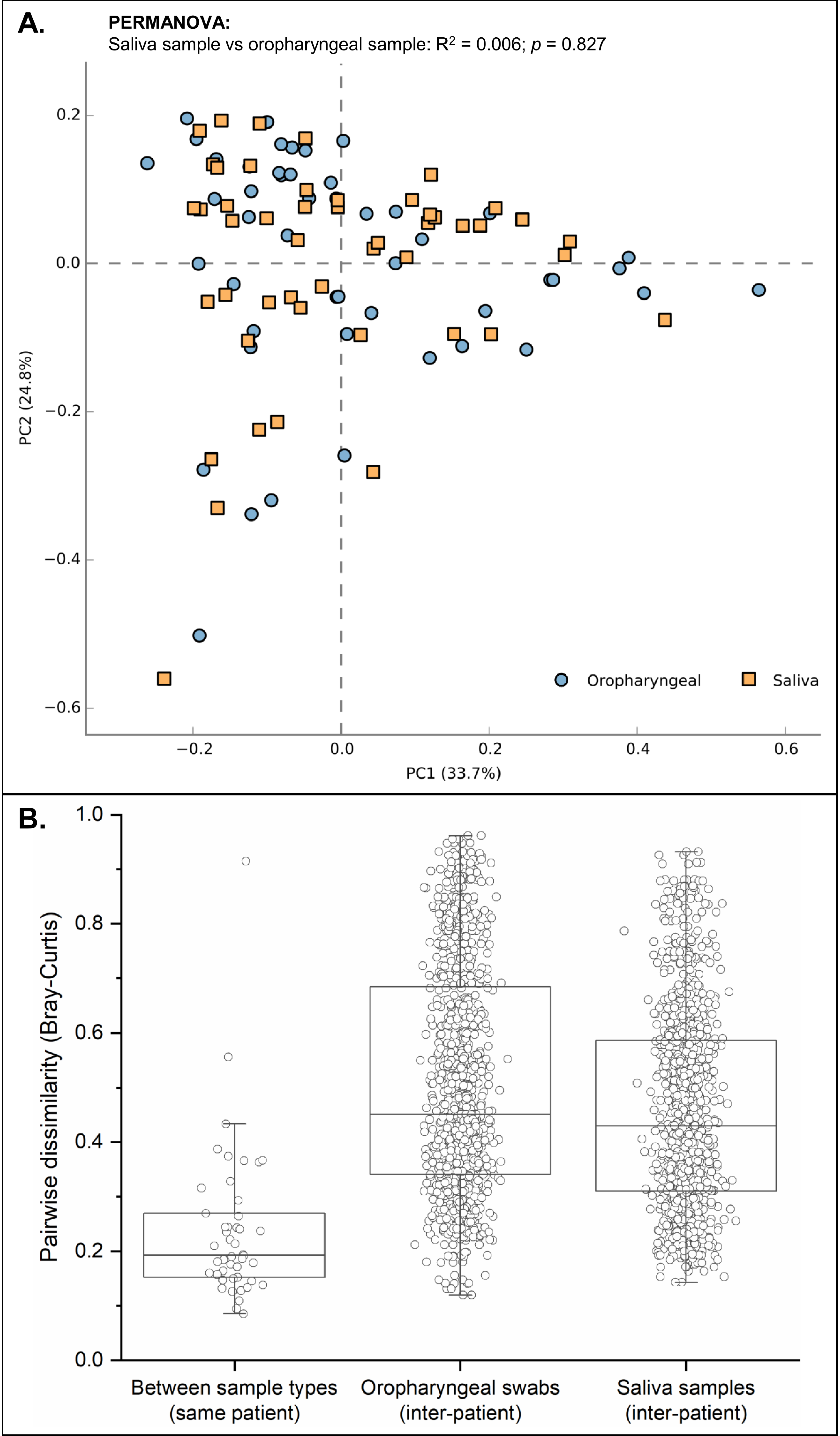
A.
PERMANOVA:
Saliva sample vs oropharyngeal sample: R2 = 0.006; p = 0.827
PC2 (24.8%)
PC1 (33.7%)
Oropharyngeal
Saliva
B.
Pairwise dissimilarity (Bray-Curtis)
Between sample types (same patient)
Oropharyngeal swabs (inter-patient)
Saliva samples (inter-patient)

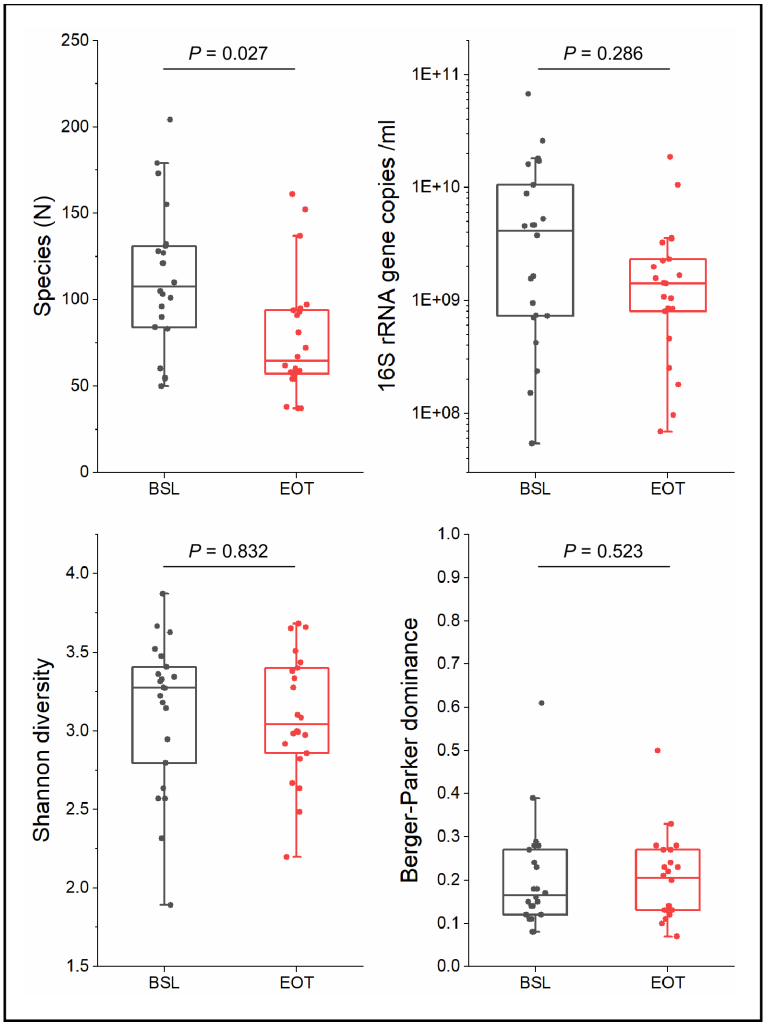

P = 0.027
Species (N)
250
200
150
100
50
0
BSL
EOT
P = 0.286
16S rRNA gene copies /ml
1E+11
1E+10
1E+09
1E+08
BSL
EOT
P = 0.832
Shannon diversity
4.0
3.5
3.0
2.5
2.0
1.5
BSL
EOT
P = 0.523
Berger-Parker dominance
1.0
0.9
0.8
0.7
0.6
0.5
0.4
0.3
0.2
0.1
0.0
BSL
EOT

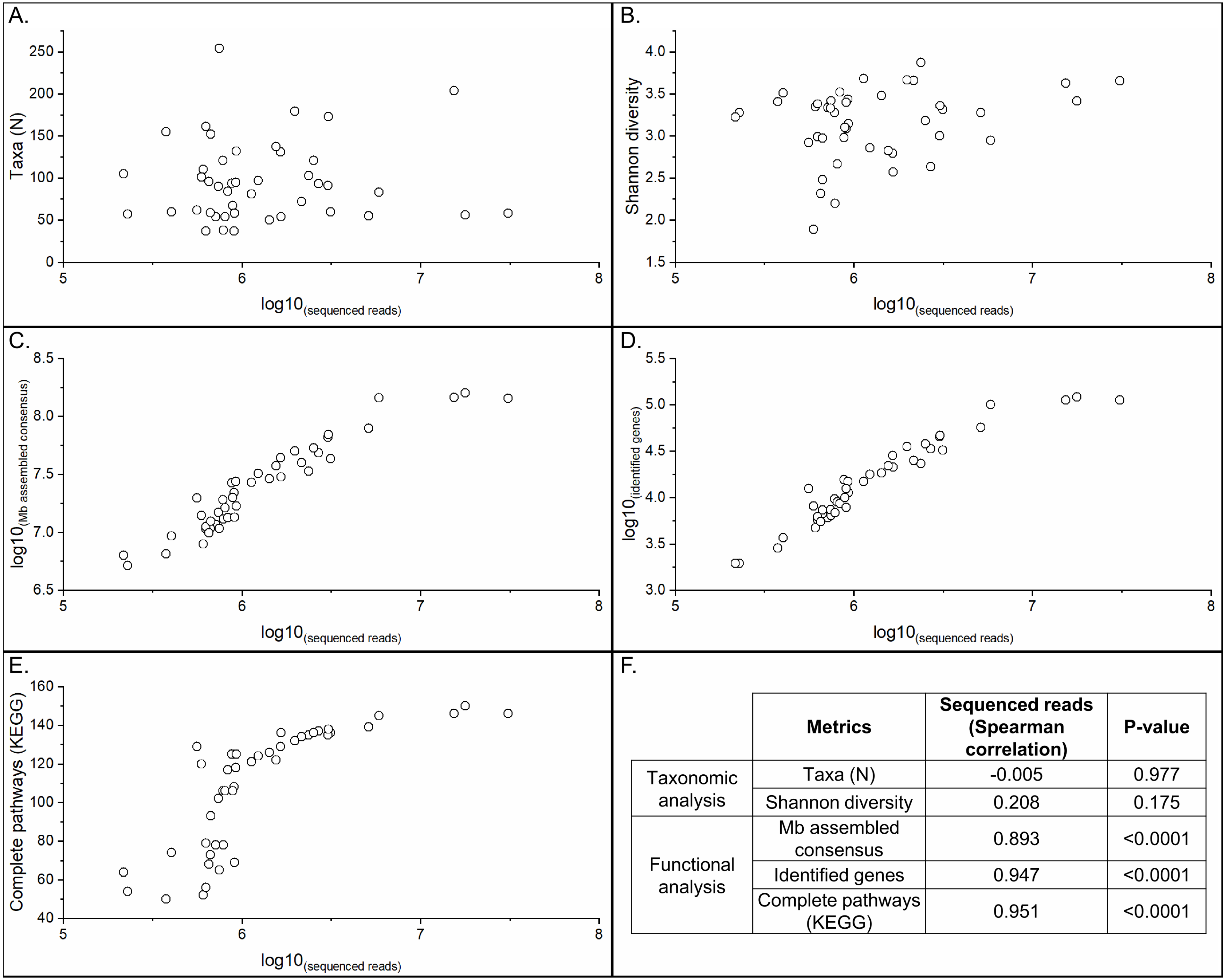

F.

	Metrics	Sequenced reads (Spearman correlation)	P-value
Taxonomic analysis	Taxa (N)	-0.005	0.977
	Shannon diversity	0.208	0.175
Functional analysis	Mb assembled consensus	0.893	<0.0001
	Identified genes	0.947	<0.0001
	Complete pathways (KEGG)	0.951	<0.0001

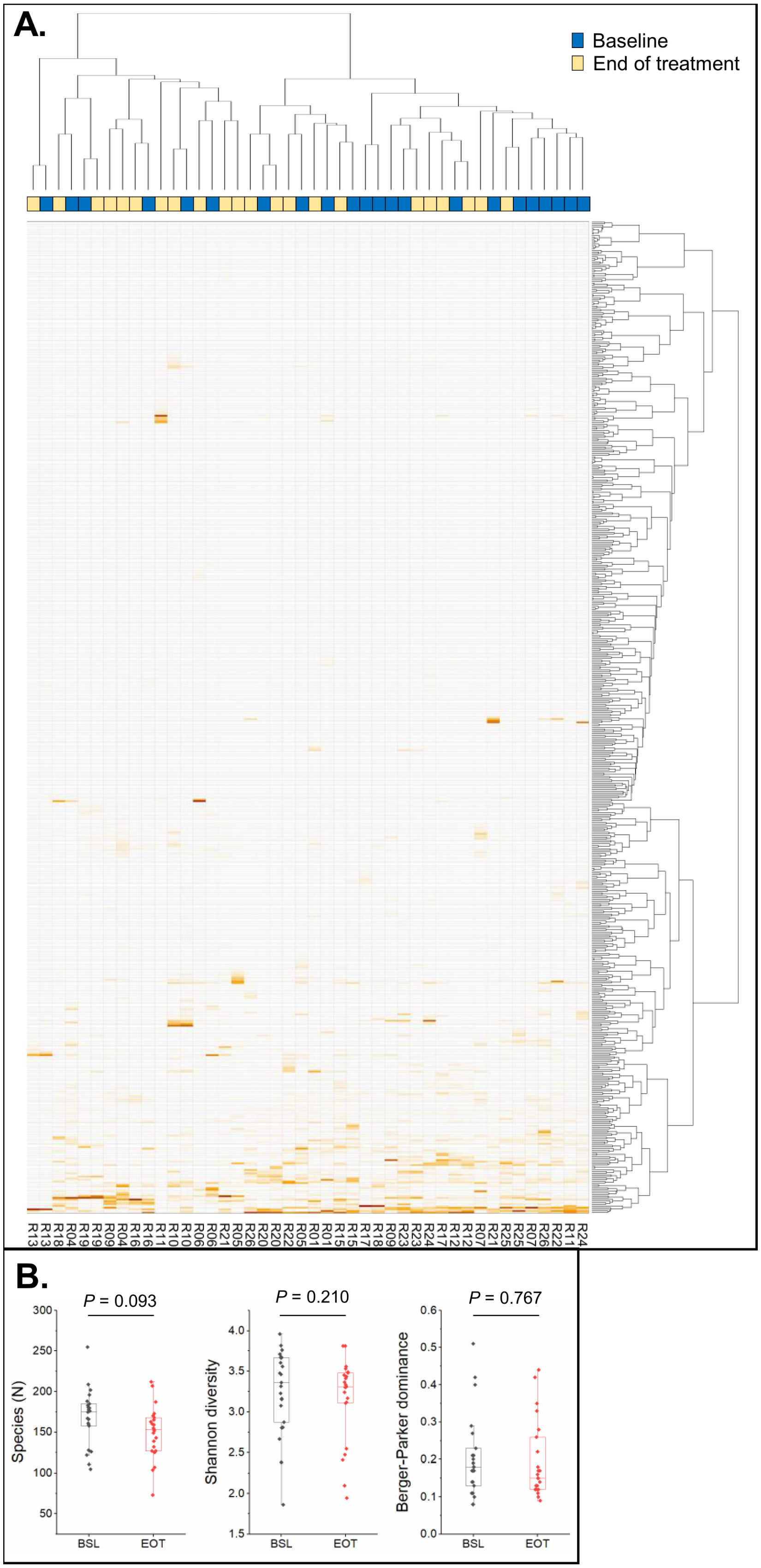
A.
Baseline
End of treatment
B.
P = 0.093
Species (N)
BSL
EOT
P = 0.210
Shannon diversity
BSL
EOT
P = 0.767
Berger-Parker dominance
BSL
EOT

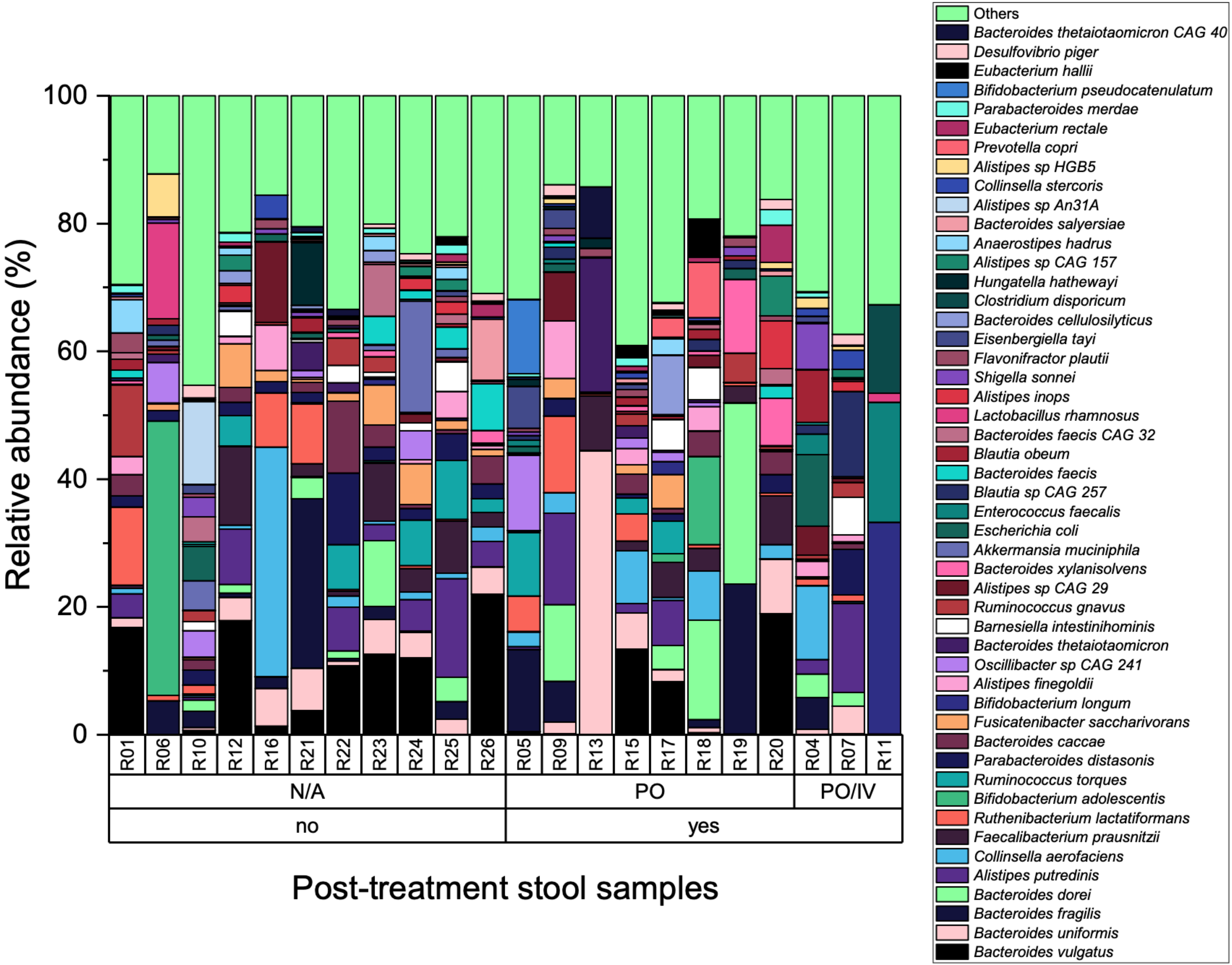
Relative abundance (%)
100
80
60
40
20
0
R01
R06
R10
R12
R16
R21
R22
R23
R24
R25
R26
R05
R09
R13
R15
R17
R18
R19
R20
R04
R07
R11
N/A
PO
PO/IV
no
yes
Post-treatment stool samples
Others
Bacteroides thetaiotaomicron CAG 40
Desulfovibrio piger
Eubacterium hallii
Bifidobacterium pseudocatenulatum
Parabacteroides merdae
Eubacterium rectale
Prevotella copri
Alistipes sp HGB5
Collinsella stercoris
Alistipes sp An31A
Bacteroides salyersiae
Anaerostipes hadrus
Alistipes sp CAG 157
Hungatella hathewayi
Clostridium disporicum
Bacteroides cellulosilyticus
Eisenbergiella tayi
Flavonifractor plautii
Shigella sonnei
Alistipes inops
Lactobacillus rhamnosus
Bacteroides faecis CAG 32
Blautia obeum
Bacteroides faecis
Blautia sp CAG 257
Enterococcus faecalis
Escherichia coli
Akkermansia muciniphila
Bacteroides xylanisolvens
Alistipes sp CAG 29
Ruminococcus gnavus
Barnesiella intestinihominis
Bacteroides thetaiotaomicron
Oscillibacter sp CAG 241
Alistipes finegoldii
Bifidobacterium longum
Fusicatenibacter saccharivorans
Bacteroides caccae
Parabacteroides distasonis
Ruminococcus torques
Bifidobacterium adolescentis
Ruthenibacterium lactatiformans
Faecalibacterium prausnitzii
Collinsella aerofaciens
Alistipes putredinis
Bacteroides dorei
Bacteroides fragilis
Bacteroides uniformis
Bacteroides vulgatus

Supplementary Table 1. Cohort characteristics.

Variable	N = 22
**Median age** (range)	61 (range 50-71)
**Sex** (%)	
Male	19 (86)
Female	3 (14)
**Smoking status** (%)	
Current	4 (17)
Former	10 (48)
Never-smoker	8 (35)
**Smoking pack-years** (%)	
<10	10 (45)
$\geq$10	12 (55)
**Primary** (%)	
Base of tongue	7 (32)
Tonsil	13 (59)
Soft palate	2 (9)
**T 8th Ed.** (%)	
T0-2	10 (46)
T3	6 (27)
T4	6 (27)
**N 8th Ed.** (%)	
N0	4 (18)
N1-2	15 (68)
N3	3 (14)
**Stage AJCC 8th ed.** (%)	
I	6 (28)
II	8 (36)
III	8 (36)
**Tooth extraction prior CRT**	
Yes	5 (23)
No	9 (41)
Unknown	8 (36)
**Radiation completion** (%)	
Yes	22 (100%)
No	0
**Cisplatin dose** (%)	
<200 mg/m^2	6 (28)
=200 mg/m^2	8 (36)
>200 mg/m^2	8 (36)
**Use of antibiotics 1-month prior or during CRT**	
Yes	11 (50%)
No	11 (50%)
**Median duration (range)**	8 days (4-15)
**Type of antibiotics**	
Penicillins	4 (36)
Cephalosporins	5 (45)
Macrolides	2 (19)
Fluorquinolones	1 (1)
**Route of antibiotic administration**	
Oral	11 (100)
Intravenous	3 (27)

**Grade 3/4 Neutropenia**<br>**Grade 3/4 Febrile Neutropenia**	4 (17)<br>1 (4)
**Grade 3/4 Mucositis**	7 (32)

Supplementary Table 2. Effect of potential confounders on the oral and stool microbiota at baseline measured using a PERMANOVA analysis.

**Oropharyngeal samples**	R-squared	*P*-value
Smoking status (Current/Former/Never)	0.084	0.498
Tumour location (Base of tongue/Soft palate/Tonsil)	0.096	0.315
Stage (I/II/III)	0.110	0.198
T-staging (T1-4)	0.180	0.062

**Stool samples**	R-squared	*P*-value
Smoking status (Current/Former/Never)	0.107	0.204
Tumour location (Base of tongue/Soft palate/Tonsil)	0.069	0.903
Stage (I/II/III)	0.089	0.519
T-staging (T1-4)	0.125	0.701

Supplementary Table 3: Effect of antibiotics on the composition in post-treatment stool samples

**Variable**	R-squared	*P*-value
Antibiotics	0.05	0.33
Route of administration	0.063	0.113

Supplementary Table 4. Interaction between potential confounders and CRT

**Oropharyngeal samples**	R-squared	*P*-value
Collection time	**0.118**	**0.001**
Collection time vs Smoking status (Current/Former/Never)	0.069	0.894
Collection time vs Tumour location (Base of tongue/Soft palate/Tonsil)	0.074	0.816
Collection time vs Stage (I/II/III)	0.076	0.755
Collection time vs T-staging (T1-4)	0.147	0.353
Collection time vs Mucositis (G1-3)	0.111	0.156
Collection time vs Antibiotics (Yes/No)	0.084	0.631
Collection time vs G-tube dependency at FU	0.047	0.407

**Stool samples**	R-squared	*P*-value
Collection time	0.034	0.146
Collection time vs Smoking status (Current/Former/Never)	0.110	0.363
Collection time vs Tumour location	0.078	0.964
Collection time vs Stage (I/II/III)	0.093	0.775
Collection time vs T-staging (T1-4)	0.121	0.956
Collection time vs Mucositis (G1-3)	0.119	0.200
Collection time vs Antibiotics (Yes/No)	0.089	0.821
Collection time vs G-tube dependency at FU	0.043	0.791

Laboratory Manual

ROMA LA-OPSCC-001

ROLE OF MICROBIOME AS A BIOMARKER IN LOCOREGIONALLY-ADVANCED OROPHARYNGEAL SQUAMOUS CELL CARCINOMA (LA-OPSCC)

Version 1.0

Prepared by
Correlative Studies Program
Princess Margaret Cancer Centre
Toronto ON

8-DEC-2017

Table of Contents

Table of Contents 2

Contacts 3

Executive Summary 4

Schedule of Events 5

Specimen Collection Procedures 6

- Saliva Specimens 7
- Oropharyngeal Swab Specimens 9
- Stool Specimens 11
- Rectal Swab Specimens 14

Appendix A – General Guidelines 16

- General Shipping Instructions 17
- Shipping within Canada 18
- Shipping with Dry Ice 19
- General Processing Guidelines 20
- Glossary of Terms 20

Appendix B – Kits 21

- Kit and Specimen Collection Supplies 22
- Kit Request Form 23

Appendix C – Requisitions 24

- SALIVA SPECIMEN REQUISITION 25
- OROPHARYNGEAL SWAB SPECIMEN REQUISITION 26
- STOOL SPECIMEN REQUISITION 27
- RECTAL SWAB SPECIMEN REQUISITION 28

Appendix D – Shipping Documents 29

- Notification of Sample Shipment 30

Appendix E – Patient Instructions 31

Appendix F – DNA/RNA Shield Instructions 33

Contacts

If you have any questions regarding specimen collection and shipping procedures, please contact the Central Laboratory, the Correlative Studies Program at the Princess Margaret Cancer Centre directly.

Central Laboratory Address

Correlative Studies Program
Princess Margaret Cancer Centre
610 University Avenue, Room 7-420
Toronto, Ontario M5G 2M9
Canada

Contact Details

For laboratory questions, please call:
Vanessa Speers
Program Manager
Correlative Studies Program
Tel: (416) 946-4501 ext. 2562
Fax: (416) 946-2048
Email: vanessa.speers@uhn.ca

For shipping questions, please call:
Correlative Studies Office
Tel: (416) 946-4501 ext. 5047
Fax: (416) 946-4431
Email: CCRUcorrelativestudies@uhn.ca

Executive Summary

The primary objective of the trial is to assess the feasibility of microbiome evaluation in LA-OPSCC patients undergoing chemoradiotherapy (CRT) using samples collected by four different methods (i.e. saliva, oropharyngeal swab over the tumor site, stool and rectal swab).

In addition, the exploratory objective is to evaluate the relationship between oral and intestinal microbiome in LA-OPSCC patients undergoing CRT.

A breakdown of studies to be performed is outlined in Table 1; a schedule of events is outlined in Table 2.

Table 1. Proposed tests for evaluation.

Test	Specimens	Laboratory
DNA extraction<br>16S rRNA amplification<br>Ilumina MiSeq sequencing	Saliva<br>Oropharyngeal Swab<br>Stool<br>Rectal Swab	Centre for the Analysis of Genome Evolution and Function (CAGEF), Dr. Guttman, University of Toronto, Ontario, Canada

Schedule of Events

Table 2. Schedule of specimen collection.

Study Timetable:	Screening Visit	Baseline[1]	End of Treatment[2]
Saliva[3]		X	X
Oropharyngeal Swab[3]		X	X
Stool[3]		X	X
Rectal Swab[3,4]		X	X

[1] Samples at baseline must be collected prior to starting any therapy including chemotherapy and radiotherapy
[2] Samples at the end of treatment will be collected within the last week of CRT, excepting the stool samples that may be collected within the last week or up to three weeks after completion of CRT.
[3] Samples do not need to be collected concurrently.
[4] Rectal swab submission is optional.

Specimen Collection Procedures

Saliva Specimens

Saliva should be collected at baseline and at the end of CRT. Samples at baseline must be collected before starting any therapy including chemotherapy and radiotherapy.

Each tube will be labeled with the protocol ID, patient initials, patient study ID, study visit, as well as date and time of specimen collection.

Fill in the Saliva Specimen Requisition at the time of collection (Appendix C).

Table 3. Saliva specimen to be collected.

Saliva Sample
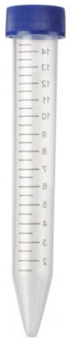
1 x 15 mL tube with DNA/RNA Shield

Saliva Processing Procedures:

1. Fill in the specimen label and affix to the 15 mL conical tube
2. Ask patient to produce a saliva sample in the petri dish provided in the Saliva Kit
3. Open the sterile disposable pipette
4. Use the pipette to transfer 1 mL of saliva into the 15 mL conical tube containing the DNA/RNA Shield reagent
    - For more information regarding the DNA/RNA Shield reagent, see Appendix F
5. Tightly close the conical tube
6. Invert 10 times to mix
7. Freeze and store at -20°C until shipment

Frozen Saliva Shipping Procedures:

1. Take the shipping box and add a one-inch layer of dry ice to the bottom of the styrofoam insert
2. Remove frozen samples from freezer. Place samples in a sealed biohazard bag

3. Place the completed requisition in the outer pocket of the biohazard bag
4. Place the sealed biohazard bag(s) containing the samples at the bottom of the styrofoam shipping box
5. Surround and cover the box(es) containing the samples with as much dry ice as possible, filling the shipping box. The shipping box should be completely full
6. Label the shipping container with appropriate labels and shipping addresses
7. Batch ship frozen every three months (Monday to Wednesday only) on dry ice to the Correlative Studies Program at The Princess Margaret Cancer Centre
8. **Ensure that the shipping box is large enough to accommodate a generous amount of dry ice to maintain the temperature of the sample(s) for the length of the transit time plus an additional 24 hours. Failure to do so can result in thawing of the specimen(s), which will render it unusable for study purposes**
9. Complete and email a copy of the Notification of Sample Shipment form (Appendix D) on the day of shipment

Shipping Address

Correlative Studies Program
Princess Margaret Cancer Centre
610 University Avenue, Room 7-420
Toronto, Ontario M5G 2M9
Canada
Tel: (416) 946-4501 ext. 5047
Fax: (416) 946-4431
Email: CCRUcorrelativestudies@uhn.ca

UHN Princess Margaret Cancer Centre

Oropharyngeal Swab Specimens

An oropharyngeal swab should be collected at baseline and at the end of CRT. Samples at baseline must be collected before starting any therapy including chemotherapy and radiotherapy.

Each tube will be labeled with the protocol ID, patient initials, patient study ID, study visit, as well as date and time of specimen collection.

Fill in the Oropharyngeal Swab Specimen Requisition at the time of collection (Appendix C).

Table 4. Oropharyngeal swab specimen to be collected.

Oropharyngeal Swab
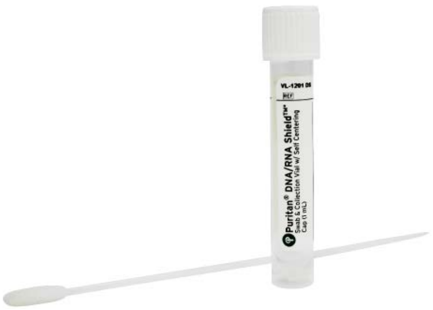
1 x DNA/RNA Shield Collection Tube

Oropharyngeal Swab Processing Procedures:

1. Open the package containing the swab and collection tube
    - For more information regarding the DNA/RNA Shield swab kit, see Appendix F
2. Peel open the swab package and remove the swab
3. Avoid touching swab tip with gloves or against any surface
4. Have the patient open their mouth and immediately bring swab tip to inside of cheek
5. Gently rub and rotate swab over the tumour site for 5-10 seconds, ensuring that the entire swab-tip has made contact with the area
6. Open the collection tube and insert swab tip into the solution
7. Break the swab leaving the swab tip in the collection tube
8. Cap and invert tube 10 times to mix
9. Freeze and store at -20°C until shipment

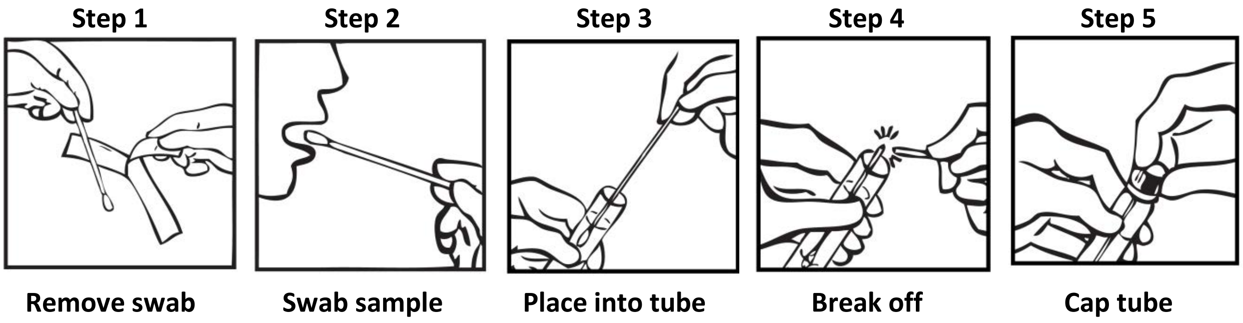

Figure 1. Oropharyngeal swab specimen collection.

Frozen Oropharyngeal Swab Shipping Procedures:

1. Take the shipping box and add a one-inch layer of dry ice to the bottom of the styrofoam insert
2. Remove frozen samples from freezer. Place samples in a sealed biohazard bag
3. Place the completed requisition in the outer pocket of the biohazard bag
4. Place the sealed biohazard bag(s) containing the samples at the bottom of the styrofoam shipping box
5. Surround and cover the box(es) containing the samples with as much dry ice as possible, filling the shipping box. The shipping box should be completely full
6. Label the shipping container with appropriate labels and shipping addresses
7. Batch ship frozen every three months (Monday to Wednesday only) on dry ice to the Correlative Studies Program at The Princess Margaret Cancer Centre
8. **Ensure that the shipping box is large enough to accommodate a generous amount of dry ice to maintain the temperature of the sample(s) for the length of the transit time plus an additional 24 hours. Failure to do so can result in thawing of the specimen(s), which will render it unusable for study purposes**
9. Complete and email a copy of the Notification of Sample Shipment form (Appendix D) on the day of shipment

Shipping Address

Correlative Studies Program
Princess Margaret Cancer Centre
610 University Avenue, Room 7-420
Toronto, Ontario M5G 2M9
Canada
Tel: (416) 946-4501 ext. 5047
Fax: (416) 946-4431
Email: CCRUcorrelativestudies@uhn.ca

Stool Specimens

Stool should be collected at baseline and at the end of CRT. Samples at baseline must be collected before starting any therapy including chemotherapy and radiotherapy. The end of treatment sample may be collected within the last week of CRT or up to three weeks after completion of CRT.

Each tube will be labeled with the protocol ID, patient initials, patient study ID, study visit, as well as date and time of specimen collection.

Fill in the Stool Specimen Requisition at the time of collection (Appendix C).

Table 5. Stool specimen to be collected.

Stool Sample
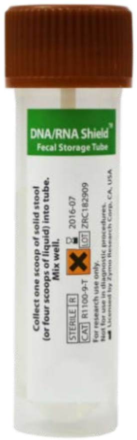
1 x DNA/RNA Shield Fecal Collection Tube

Stool Processing Procedures:

1. Fill in patient information on the label and affix to the collection tube
2. Affix date and time label to the tube. Patient should fill in this label once sample is collected
3. Provide patient with collection supplies from the Stool Kit:
   - 1 x Patient instruction sheet (Appendix E)
   - 1 x Commode specimen collector
   - 1 x Labeled DNA/RNA Shield fecal collection tube (also see Appendix F)
   - 1 x Biohazard bag
4. Review the following instructions with patient:
   - Place commode specimen collector under toilet seat

- Void bowel movement into commode specimen collector
- To prevent contamination, the fecal sample must not come into contact with toilet water
- Unscrew the cap of the collection tube
- Use the spoon to scoop one spoonful of feces from the commode specimen collector (approximately 1 gram or 1 mL)
- Place sample into the collection tube
- Tighten the cap
- Shake 10 times to mix the contents thoroughly
- Some fecal material may be difficult to re-suspend. As long as the material is suspended, the sample is stabilized. Foaming/frothing during shaking is normal.
- Dispose of unused fecal material and thoroughly wash hands
- Record date and time of collection on the label

5. Patient should return sample to clinic within three-five days
6. Complete the Stool Specimen Requisition (Appendix C)
7. Freeze and store at -20°C until shipment

Frozen Stool Shipping Procedures:

1. Take the shipping box and add a one-inch layer of dry ice to the bottom of the styrofoam insert.
2. Remove frozen samples from freezer. Place samples in a sealed biohazard bag.
3. Place the completed requisition in the outer pocket of the biohazard bag.
4. Place the sealed biohazard bag(s) containing the samples at the bottom of the styrofoam shipping box.
5. Surround and cover the box(es) containing the samples with as much dry ice as possible, filling the shipping box. The shipping box should be completely full.
6. Label the shipping container with appropriate labels and shipping addresses.
7. Batch ship frozen every three months (Monday to Wednesday only) on dry ice to the Correlative Studies Program at The Princess Margaret Cancer Centre.
8. **Ensure that the shipping box is large enough to accommodate a generous amount of dry ice to maintain the temperature of the sample(s) for the length of the transit time plus an additional 24 hours. Failure to do so can result in thawing of the specimen(s), which will render it unusable for study purposes.**
9. Complete and email a copy of the Notification of Sample Shipment form (Appendix D) on the day of shipment.

Shipping Address
Correlative Studies Program
Princess Margaret Cancer Centre
610 University Avenue, Room 7-420
Toronto, Ontario M5G 2M9
Canada
Tel: (416) 946-4501 ext. 5047
Fax: (416) 946-4431
Email: CCRUcorrelativestudies@uhn.ca

Rectal Swab Specimens

Rectal swab submission is optional. For consenting patient, a rectal swab should be collected at baseline and at the end of CRT. Samples at baseline must be collected before starting any therapy including chemotherapy and radiotherapy.

Each tube will be labeled with the protocol ID, patient initials, patient study ID, study visit, as well as date and time of specimen collection.

Fill in the Rectal Swab Specimen Requisition at the time of collection (Appendix C).

Table 4. Rectal swab specimen to be collected.

Rectal Swab
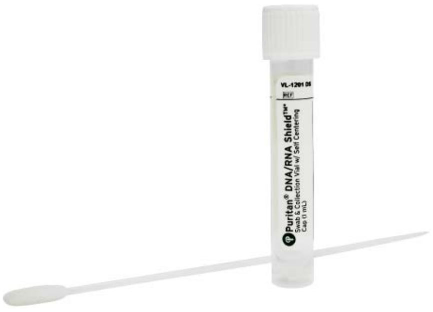
1 x DNA/RNA Shield Collection Tube

Rectal Swab Processing Procedures:

1. Open the package containing the swab and collection tube
    - For more information regarding the DNA/RNA Shield swab kit, see Appendix F
2. Peel open the swab package and remove the swab
3. Avoid touching swab tip with gloves or against any surface
4. Do not lubricate the swab. If necessary, sterile saline may be used to moisten the swab
5. Insert the swab approximately one inch into the anal canal
6. Rotate the swab slowly for 10 seconds then remove
7. Open the collection tube and place swab into the solution
8. Break the swab leaving the swab tip in the collection tube
9. Cap and invert tube 10 times to mix
10. Freeze and store at -20°C until shipment

Frozen Rectal Swab Shipping Procedures:

1. Take the shipping box and add a one-inch layer of dry ice to the bottom of the styrofoam insert
2. Remove frozen samples from freezer. Place samples in a sealed biohazard bag
3. Place the completed requisition in the outer pocket of the biohazard bag
4. Place the sealed biohazard bag(s) containing the samples at the bottom of the styrofoam shipping box
5. Surround and cover the box(es) containing the samples with as much dry ice as possible, filling the shipping box. The shipping box should be completely full
6. Label the shipping container with appropriate labels and shipping addresses
7. Batch ship frozen every three months (Monday to Wednesday only) on dry ice to the Correlative Studies Program at The Princess Margaret Cancer Centre
8. **Ensure that the shipping box is large enough to accommodate a generous amount of dry ice to maintain the temperature of the sample(s) for the length of the transit time plus an additional 24 hours. Failure to do so can result in thawing of the specimen(s), which will render it unusable for study purposes**
9. Complete and email a copy of the Notification of Sample Shipment form (Appendix D) on the day of shipment

Shipping Address

Correlative Studies Program
Princess Margaret Cancer Centre
610 University Avenue, Room 7-420
Toronto, Ontario M5G 2M9
Canada
Tel: (416) 946-4501 ext. 5047
Fax: (416) 946-4431
Email: CCRUcorrelativestudies@uhn.ca

Appendix A – General Guidelines

General Shipping Instructions

Frozen samples: ship Monday through Wednesday ONLY

Prior to shipping, please refer to the observed holiday schedule below

Complete the 'Notification of Sample Shipment Form' with all the necessary information (Appendix D)

Email a completed 'Notification of Sample Shipment Form' to the Correlative Studies Program on the day the shipment is sent

Address sample shipments to the attention of:

Correlative Studies Program
Princess Margaret Cancer Centre
610 University Ave 7-420
Toronto, ON
M5G 2M9
Tel: 416-946-4501 ex: 5047
Fax: 416-946-4431
CCRUcorrelativestudies@uhn.ca

Observed Holidays*
New Year's Day
Family Day
Good Friday
Easter Monday
Victoria Day
Canada Day
Civic Holiday
Labor Day
Thanksgiving
Christmas Day
Boxing Day

* Please ensure that shipments are not scheduled to arrive on the observed holidays (Ontario, Canada) listed in the table. For exact holiday dates, please refer to FedEx link: http://www.fedex.com/ca_english/services/serviceguide/holidays.html, or your local courier provider. Service (pick-up and delivery) may be limited prior to, during and following observed holidays in which you are shipping specimens.

Shipping within Canada

Shipments can be delayed if documentation is not complete. For ease of delivery, we strongly recommend that you use FedEx. Please ensure the following is complete prior to sending your shipment to the Princess Margaret in Toronto.

When shipping within Canada, please make sure to use a FedEx Domestic Waybill. For step-by-step instructions on how to complete the Intra-Canada Waybill using the online FedEx Ship Manager, please go to:

http://www.fedex.com/ca_english/helpguide/Published/FedExCanada/HTML/Content/CA_Domestic_Shipping.pdf

Key Points:

1. Sender Information - Enter your shipping information. This includes the address you are shipping from, your name, your phone number and your FedEx account number. If you need a FedEx account number, register for one now.

2. Recipient Information- Complete the details for your shipping destination. This includes recipient name, address and phone number.

3. Service - Indicate which service you are using. Select FedEx Priority Overnight.

4. Packaging - Indicate the FedEx Express packaging you are using, or if you are using your own packaging.

5. Billing – The sender must cover all shipping fees. Enter your FedEx account number. You may assign internal billing numbers or codes here. The first 24 characters will appear in your invoice.

6. Special Services – Mark "Yes" or "No" to indicate whether your shipment contains dangerous goods (i.e. dry ice). If so, mark "Yes as per attached Shipper's Declaration," and include three copies of a Shipper's Declaration for Dangerous Goods. Restrictions apply for dangerous goods — see the FedEx Express Terms and Conditions.

If your shipment contains dry ice (UN 1845), mark the dry ice box and list the number of packages and net quantity per package of dry ice in kilograms

Shipping with Dry Ice

Dry ice is classified by the International Air Transport Association (IATA) as a "miscellaneous" hazard, class 9. Dry ice is considered hazardous during transportation for three reasons:

Explosion hazard: dry ice releases a large volume of carbon dioxide gas as it sublimates. If packaged in a container that does not allow for release of the gas, it may explode, causing personal injury or property damage.

Suffocation hazard: a large volume of carbon dioxide gas emitted in a confined space may create an oxygen deficient atmosphere.

Contact hazard: dry ice is a cryogenic material that causes severe frostbite upon contact with skin. Personal protective equipment should be used when handling dry ice.

Packaging dry ice properly will minimize the risk to personnel transporting the material. The explosion hazard will be eliminated with a package designed to vent gaseous carbon dioxide. Suffocation and contact hazards will be greatly reduced by labeling the package correctly, so those who come in contact with it will be aware of the contents. Only personnel that have received their Transportation of Dangerous Goods can ship packages containing dry ice.

General Processing Guidelines

Remember to follow the instructions regarding storage and shipping of the samples after they have been collected. Use appropriate personal protective equipment and thoroughly wash hands according to your institution's guidelines.

Glossary of Terms

CAGEF – Centre for the Analysis of Genome Evolution and Function

CCRU - Cancer Clinical Research Unit

CRT – Chemoradiotherapy

DNA – Deoxyribonucleic acid

LA-OPSCC – Locoregionally-Advanced Oropharyngeal Squamous Cell Cancer

RNA – Ribonucleic acid

rRNA – Ribosomal RNA

RT – Radiotherapy

<u>Collection Tubes</u>

DNA/RNA Shield (see Appendix F)

Appendix B – Kits

Kit and Specimen Collection Supplies

The following is being supplied as kits for this study:

Name	Kit components	Notes
Saliva Kit	1 x Petri dish<br>1 x 15 mL conical tube with 1 mL DNA/RNA Shield reagent<br>1 x Sterile disposable pipette<br>1 x Label<br>1 x Biohazard bag	Do not use supplies after expiry date
Oral Swab Kit	1 x DNA/RNA Shield Collection Vial with sterile swab<br>1 x Label<br>1 x Biohazard bag	Do not use supplies after expiry date
Stool Kit	1 x Patient instruction sheet<br>1 x Commode specimen collector<br>1 x DNA/RNA Shield Fecal Collection Tube<br>2 x Labels<br>1 x Biohazard bag	Do not use supplies after expiry date
Rectal Swab Kit	1 x DNA/RNA Shield Collection Vial with sterile swab<br>1 x Label<br>1 x Biohazard bag	Do not use supplies after expiry date

For University Health Network (UHN) staff, please reorder supplies through the CCRU Kit Squad storefront. To obtain storefront access, email CCRUkitsquad@uhn.ca.

For all other non-UHN sites, please reorder supplies using the Kit Supply Request Form in Appendix B of this lab manual and email it to the CCRU Kit Squad: CCRUkitsquad@uhn.ca

Princess Margaret Cancer Centre

Kit Request Form

***Order supplies at least 10 business days in advance ***

Protocol	ROMA LA-OPSCC-001	Site	
Date Ordered (dd/mmm/yyyy)	__ __/__ __ __/__ __ __ __	Date Required (dd/mmm/yyyy)	__ __/__ __ __/__ __ __ __
Requestor Name		Requestor Email Address	

Kits to Request		
Kit:	Quantity	Office Use Only
Saliva Kit		
Oral Swab Kit		
Stool Kit		
Rectal Swab Kit		

Shipping Address

For Office Use Only			
Received by		Date	__ __/__ __ __/__ __ __ __
Completed by		Date	__ __/__ __ __/__ __ __ __
Courier		Date	__ __/__ __ __/__ __ __ __
Waybill Number			
Date Delivered	__ __/__ __ __/__ __ __ __		

EMAIL TO: CCRUkitsquad@uhn.ca

Appendix C – Requisitions

INSTRUCTIONS: Initial institution should complete all information below. **Email the form in advance of shipping the specimen.** In all cases, a copy of the form should be transported with the specimen. The original should be sent to the study coordinator.

SALIVA SPECIMEN REQUISITION

Protocol No.	ROMA LA-OPSCC-001	Site Name	
Pt. Initials	____ - ____ - ____ (F-M-L)	Subject ID	
CRA Completing this Form (print)		CRA Email Address	

Study Time point *(Check one)*	***Use a new requisition for each time point collected***
☐ Baseline	☐ End of CRT

**Date (DD-MMM-YYYY)**	**Time (24 hour clock)**	**Collection Tube**
		1 x 15 mL tube with DNA/RNA Shield
Were the subject's gums bleeding at the time of collection? ☐ Yes ☐ No		

**Comments**

**Shipping Information**	
Courier	
Waybill Number	
Shipment Date	__ __/__ __ __/__ __ __ __ (dd/mmm/yyyy)
CRA Shipping Samples	
CRA Email Address	

**For Office Use Only**	
Specimen Received by	
Date Received	__ __/__ __ __/__ __ __ __ (dd/mmm/yyyy)

INSTRUCTIONS: Initial institution should complete all information below. **Email the form in advance of shipping the specimen.** In all cases, a copy of the form should be transported with the specimen. The original should be sent to the study coordinator.

OROPHARYNGEAL SWAB SPECIMEN REQUISITION

Protocol No.	ROMA LA-OPSCC-001	Site Name	
Pt. Initials	____ - ____ - ____ (F-M-L)	Subject ID	
CRA Completing this Form (print)		CRA Email Address	

Study Time point *(Check one)*	***Use a new requisition for each time point collected***
☐ Baseline	☐ End of CRT

**Date (DD-MMM-YYYY)**	**Time (24 hour clock)**	**Collection Tube**
		1 x DNA/RNA Shield Collection Vial

**Comments**

**Shipping Information**	
Courier	
Waybill Number	
Shipment Date	__ __/__ __ __/__ __ __ __ (dd/mmm/yyyy)
CRA Shipping Samples	
CRA Email Address	

**For Office Use Only**	
Specimen Received by	
Date Received	__ __/__ __ __/__ __ __ __ (dd/mmm/yyyy)

INSTRUCTIONS: Initial institution should complete all information below. **Email the form in advance of shipping the specimen.** In all cases, a copy of the form should be transported with the specimen. The original should be sent to the study coordinator.

STOOL SPECIMEN REQUISITION

Protocol No.	ROMA LA-OPSCC-001	Site Name	
Pt. Initials	____ - ____ - ____ (F-M-L)	Subject ID	
CRA Completing this Form (print)		CRA Email Address	

Study Time point *(Check one)*	***Use a new requisition for each time point collected***
☐ Baseline	☐ End of CRT

**Date (DD-MMM-YYYY)**	**Time (24 hour clock)**	**Collection Tube**
		1 x DNA/RNA Shield Fecal Collection Tube

**Comments**

**Shipping Information**	
Courier	
Waybill Number	
Shipment Date	__ __/__ __ __/__ __ __ __ (dd/mmm/yyyy)
CRA Shipping Samples	
CRA Email Address	

**For Office Use Only**	
Specimen Received by	
Date Received	__ __/__ __ __/__ __ __ __ (dd/mmm/yyyy)

INSTRUCTIONS: Initial institution should complete all information below. **Email the form in advance of shipping the specimen.** In all cases, a copy of the form should be transported with the specimen. The original should be sent to the study coordinator.

RECTAL SWAB SPECIMEN REQUISITION

Protocol No.	ROMA LA-OPSCC-001	Site Name	
Pt. Initials	_____ - _____ - _____ (F-M-L)	Subject ID	
CRA Completing this Form (print)		CRA Email Address	

Study Time point *(Check one)*	***Use a new requisition for each time point collected***
☐ Baseline	☐ End of CRT

**Date (DD-MMM-YYYY)**	**Time (24 hour clock)**	**Collection Tube**
		1 x DNA/RNA Shield Collection Vial

**Comments**

**Shipping Information**	
Courier	
Waybill Number	
Shipment Date	__ __/__ __ __/__ __ __ __ (dd/mmm/yyyy)
CRA Shipping Samples	
CRA Email Address	

**For Office Use Only**	
Specimen Received by	
Date Received	__ __/__ __ __/__ __ __ __ (dd/mmm/yyyy)

Appendix D – Shipping Documents

Notification of Sample Shipment

**Shipments within Canada**
☐ Email CCRUcorrelativestudies@UHN.ca<br>☐ Attach this form<br>☐ Attach copy of specimen requisition<br>☐ Attach copy of the air waybill

Protocol #	ROMA LA-OPSCC-001
To:	Correlative Studies Program, Princess Margaret Cancer Centre
From:	
Site	
Sender Name	
Phone #	
Email	

Sample Information – Please indicate shipment contents				
Sample Type	☐ Saliva	☐ Oral Swab	☐ Stool	☐ Rectal Swab
Number of Samples	Tubes:____	Tubes:____	Tubes:____	Tubes:____
Storage Conditions	☐ Frozen	☐ Frozen	☐ Frozen	☐ Frozen

Shipping Information	
Courier	
Waybill Number	
Shipment Date	__ __/__ __ __/__ __ __ __ (dd/mmm/yyyy)

For Office Use Only	
Specimen Received by	
Date Received	__ __/__ __ __/__ __ __ __ (dd/mmm/yyyy)

Appendix E – Patient Instructions

How to Use the "POOP" Kit

Toronto General
Toronto Western
Princess Margaret
Toronto Rehab

START

STEP 1

- Collect the poop using supplies from bag
- Place collection bowl into toilet
- Poop into collection bowl
- Remove poop sample from bowl and place into collection tube.
- Prevent the poop from touching the toilet water

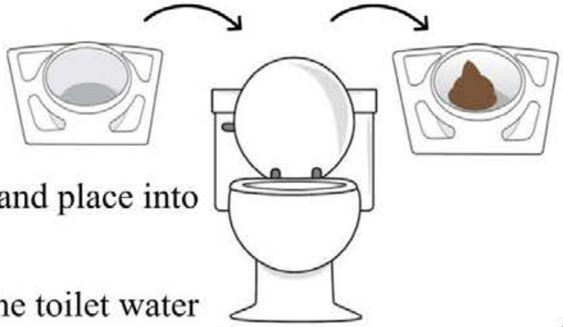

STEP 2

- Open the collection tube provided
- Use the spoon to scoop one spoonful of poop from the bowl into the tube

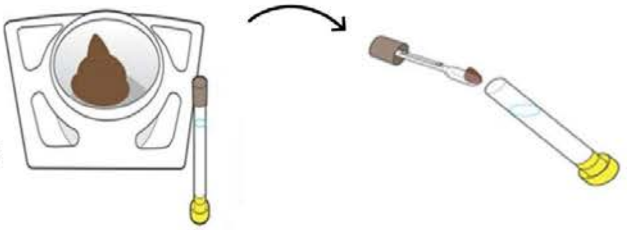

STEP 3

- Close and tighten the cap of the tube
- Shake to mix the sample
- It is OK to have some foam during and after shaking the sample

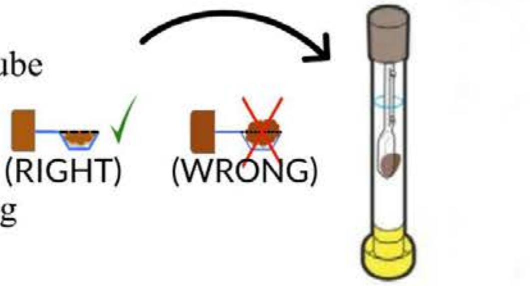

STEP 4

- Flush the rest of the poop down the toilet
- Wash your hands very well with soap and water

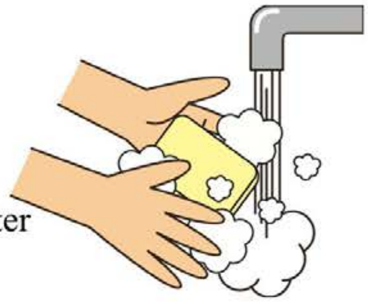

FINISH

Appendix F – DNA/RNA Shield Instructions

DNA/RNA Shield™

**Catalog Nos:**	R1100-50	(50 ml)	
	R1100-250	(250 ml)	
	R1200-25	(25 ml)	2X concentrate
	R1200-125	(125 ml)	2X concentrate
Storage:	Room Temperature		

ZYMO RESEARCH
The Beauty of Science is to Make Things Simple

Features

- ✓ Preserves genetic integrity and expression profiles of samples *(cells, tissues, blood, biological liquids, stool, etc.)* at ambient temperatures.
- ✓ DNA and RNA can be isolated directly without precipitation or reagent removal *(compatible with most DNA and RNA purification kits)*.
- ✓ Inactivates infectious agents *(virus, bacteria, yeast)*.

Description

DNA/RNA Shield™ ensures nucleic acid stability during sample storage/transport at ambient temperatures. There is no need for refrigeration or specialized equipment. DNA/RNA Shield™ effectively lyses cells and inactivates nucleases and infectious agents (virus), and it is compatible with various collection and storage devices (vacuum tubes, swabs, nasal, buccal, fecal, etc.).

Instructions for Sample Storage

In an appropriate tube or collection device, add DNA/RNA Shield™ according to the instructions below and homogenize:

Product	Recommended usage
**DNA/RNA Shield™**<br>R1100-50 (50 ml)<br>R1100-250 (250 ml)	**All samples** (cells, tissue, swabs, stool *etc.*)
	Samples should not exceed 10% (v/v).
**DNA/RNA Shield™**<br>**2X concentrate**<br>R1200-25 (25 ml)<br>R1200-125 (125 ml)	**Blood & other liquid samples**
	Mix an equal volume reagent and sample.

Store and/or transport samples in DNA/RNA Shield™ for later purification of high-quality DNA and/or RNA.

Temperature	Time
4°C - 25°C (ambient)	Up to 30 days
37°C	Up to 3 days
-20°C and below	>1 year

DNA/RNA Purification

Purify samples in DNA/RNA Shield™ directly with Zymo's DNA or RNA purification kits. Bind, wash, and then elute the DNA and/or RNA.

	Product	Size	Cat. No.
RNA	*Quick*-RNA™ Kits	50 preps<br>200 preps	R1050, R1054, R1057<br>R1051, R1055, R1058
DNA	*Quick*-DNA™ Universal Kits	50 preps<br>200 preps	D4068<br>D4069
DNA & RNA	ZR-*Duet*™ Kit	50 preps	D7001

Samples in DNA/RNA Shield™ can also be processed with other commercially available DNA and RNA purification kits.

DNA/RNA Preservation

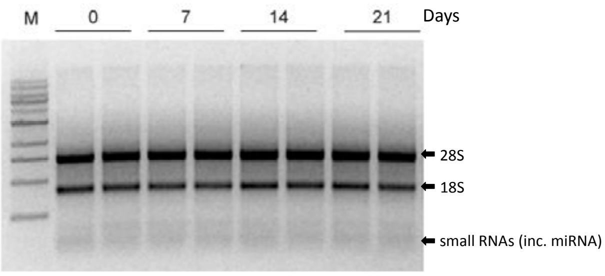

RNA from cells is effectively stabilized in DNA/RNA Shield™ at ambient temperature. Data show RNA from human cells (HCT 116) purified at the indicated time points and visualized on agarose gel.

Microbial Inactivation

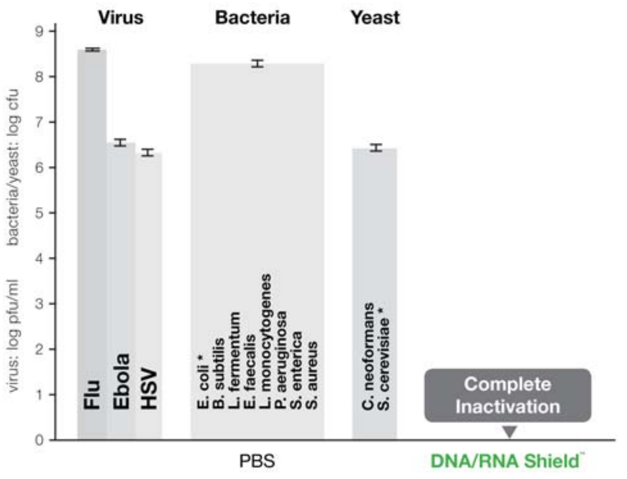

Viruses, bacteria and yeast are effectively inactivated by DNA/RNA Shield™. Samples containing the infectious agent (virus, bacteria, yeast) were treated with DNA/RNA Shield or mock (PBS) treated for 5 minutes. Titer was subsequently determined by plaque assay (PFU) or growth assay (CFU). Validated by: Influenza A – D. Poole and Prof. A. Mehle, Department of Medical Microbiology and Immunology, University of Wisconsin, Madison; Ebola (Kikwit) – L. Avena and Dr. A. Griffiths, Department of Virology and Immunology, Texas Biomedical Research Institute; HSV-1/2 – H. Oh, F. Diaz and Prof. D. Knipe, Virology Program, Harvard Medical School; E. coli*, B. subtilis, L. fermentum, E. faecalis, L. monocytogenes, P. aeruginosa, S. enteric, S. aureus, C. neoformans, S. cerevisiae* – Zymo Research Corporation). Bacterial and yeast sample inputs were 10^8-10^9 and 10^7-10^8, respectively.

Trademarks and Disclaimers
™ Trademarks of Zymo Research Corporation. Some technologies included in this product are patent pending.
This product is for research use only and should only be used by trained professionals. It is not intended for use in diagnostic applications. Wear protective gloves and eye protection. Follow the safety guidelines and rules enacted by your research institution or facility. **DNA/RNA Shield™ is available for OEM and integration into sample collection technologies.**

ZYMO RESEARCH CORP.
Phone: (949) 679-1190 ▪ Toll Free: (888) 882-9682 ▪ Fax: (949) 266-9452 ▪ info@zymoresearch.com ▪ www.zymoresearch.com

DNA/RNA Shield™ Collection Tube w/ Swab

Catalog No. R1106 (1 mL fill;10 pack) & R1107 (1 mL fill;50 pack)
Catalog No. R1108 (2 mL fill;10 pack) & R1109 (2 mL fill;50 pack)

ZYMO RESEARCH
The Beauty of Science is to Make Things Simple

Quick Protocol

Description

The DNA/RNA Shield™ Collection Tube ensures sample stability during storage/transport at ambient temperatures without a need for refrigeration or specialized equipment. DNA/RNA Shield™ reagent effectively inactivates pathogens (e.g., virus, bacteria) in samples collected with the provided swab.
Each collection tube is pre-filled with 1 mL (R1106, R1107) or 2mL (R1108, R1109) of DNA/RNA Shield™ and the nucleic acid content of samples is preserved at ambient temperature (DNA>1 year; RNA up to 1 month). Samples in DNA/RNA Shield™ can be frozen (-20/-80°C) for prolonged periods.

Instructions for use (see diagram below)

1. Open package containing swab and collection tube.
2. Peel open the swab package and remove swab.
3. Swab the sample. Common samples can include surfaces, feces, cheeks (buccal), etc.
4. Open the collection tube and insert swab tip into the solution.
5. Break the swab tip leaving the swab tip in the collection tube.
6. Cap and invert tube several times. The sample is stabilized and ready for transport/storage prior to purification of DNA and/or RNA.

Step 1

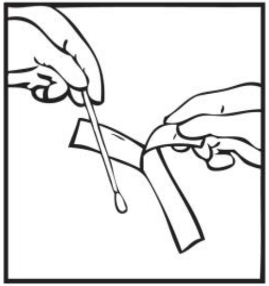

Remove swab from package

Step 2

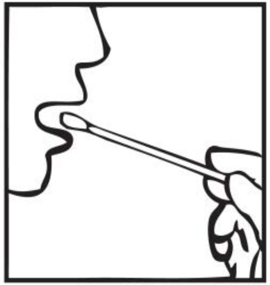

Swab sample

Step 3

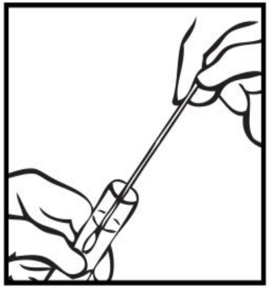

Place swab into tube

Step 4

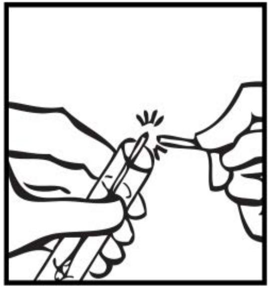

Break off swab

Step 5

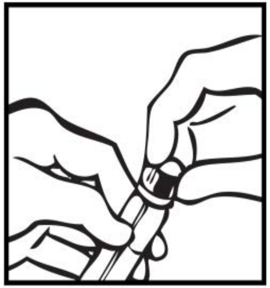

Cap tube containing sample

Warnings: DO NOT insert swab into DNA/RNA Shield™ solution before collecting a sample. DO NOT drink, touch or remove the DNA/RNA Shield™ solution from the collection tube. The DNA/RNA Shield solution can be harmful if ingested and may cause irritation if exposed to the skin and eyes. For specifics, consult product Safety Data Sheet (SDS).

DNA/RNA Shield™ Fecal Collection Tube

Catalog No. R1101

Quick Protocol

ZYMO RESEARCH
The Beauty of Science is to Make Things Simple

Description

The DNA/RNA Shield™ Fecal Collection Tube ensures sample stability during storage/transport at ambient temperatures without the need for refrigeration or specialized equipment. DNA/RNA Shield™ reagent effectively lyses samples and inactivates pathogens (e.g., virus, bacteria).

Each collection tube (with a spoon attached to the cap) is pre-filled with DNA/RNA Shield™ (9 mL). The nucleic acids (DNA & RNA) in samples are preserved at ambient temperature (DNA >1 year, RNA up to 1 month). Samples in the DNA/RNA Shield™ can be frozen (-20/-80°C) for prolonged storage.

Required Fecal Collection Accessories (Not included)

1. Fecal specimen collector set (e.g., hat-style specimen collector)
2. Labels for identification of samples
3. Appropriate waste container/biological waste container

Instructions

1. Prepare and collect fecal specimen using preferred fecal specimen collection set/kit.

   Note: Method of collecting the fecal sample must prevent feces from falling into toilet water to avoid sample contamination.

2. Unscrew the collection tube cap and use the spoon to scoop **one spoonful** of feces (approximately 1 gram or 1 mL in volume) from a sample.
3. Place the sample in the collection tube.
4. Tighten the cap and shake to mix the contents thoroughly (invert 10 times) to create a suspension.

   Note: Some fecal material may be difficult to re-suspend. As long as the material is suspended, the sample is stabilized. foaming/frothing during shaking is normal.

5. Dispose of unused fecal material and thoroughly wash hands according to your institution's guidelines.

Sample Purification

Samples in DNA/RNA Shield™ can be input directly into Zymo Research's (and others) nucleic acid purification kits.

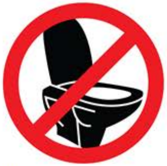

Don't let the sample go into the toilet

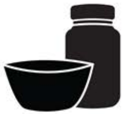

Collect stool into a clean container

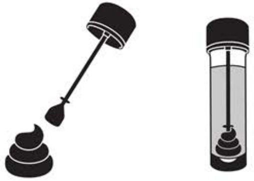

Scoop a portion of the stool sample into the DNA/RNA Shield™ Fecal Collection Tube

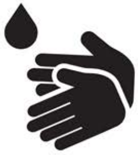

Wash hands well
